# Multiple Origins of Sex Chromosomes in *Nothobranchius* Killifishes

**DOI:** 10.1111/mec.70029

**Published:** 2025-07-24

**Authors:** Monika Hospodářská, Pablo Mora, Anna Chung Voleníková, Ahmed Al‐Rikabi, Marie Altmanová, Sergey A. Simanovsky, Nikolas Tolar, Tomáš Pavlica, Karolína Janečková, Jana Štundlová, Kseniya Bobryshava, Marek Jankásek, Matyáš Hiřman, Thomas Liehr, Martin Reichard, Eugene Yu. Krysanov, Petr Ráb, Christoph Englert, Petr Nguyen, Alexandr Sember

**Affiliations:** ^1^ Laboratory of Fish Genetics, Institute of Animal Physiology and Genetics Czech Academy of Sciences Liběchov Czech Republic; ^2^ University of South Bohemia Faculty of Science České Budějovice Czech Republic; ^3^ Institute of Entomology Biology Centre of the Czech Academy of Sciences České Budějovice Czech Republic; ^4^ Department of Experimental Biology, Genetics Area University of Jaen Jaén Spain; ^5^ Jena University Hospital, Friedrich Schiller University Institute of Human Genetics Jena Germany; ^6^ Department of Ecology, Faculty of Science Charles University Prague Czech Republic; ^7^ Severtsov Institute of Ecology and Evolution Russian Academy of Sciences Moscow Russia; ^8^ Department of Zoology, Faculty of Science Charles University Prague Czech Republic; ^9^ Department of Genetics and Microbiology, Faculty of Science Charles University Prague Czech Republic; ^10^ Institute of Computer Science Masaryk University Brno Czech Republic; ^11^ Institute of Vertebrate Biology Czech Academy of Sciences Brno Czech Republic; ^12^ Department of Ecology and Vertebrate Zoology University of Lodz Lodz Poland; ^13^ Department of Botany and Zoology, Faculty of Science Masaryk University Brno Czech Republic; ^14^ Leibniz Institute on Aging Fritz Lipmann Institute Jena Germany; ^15^ Institute of Biochemistry and Biophysics Friedrich‐Schiller‐University Jena Jena Germany

**Keywords:** bacterial artificial chromosome, chromosome fusion, pool‐seq, recombination suppression, sex chromosome differentiation, zoo‐FISH

## Abstract

Sex chromosomes have evolved repeatedly across eukaryotes. The emergence of a sex‐determining (SD) locus is expected to progressively restrict recombination, driving convergent molecular differentiation. However, evidence from taxa like teleost fishes, representing over half of vertebrate species with unmatched diversity in SD systems, challenges this model. Teleost sex chromosomes are often difficult to detect as they experience frequent turnovers, resetting the differentiation process. *Nothobranchius* killifishes, which include the XY system shared by *N. furzeri* and *N. kadleci* and X_1_X_2_Y systems in six other species, offer a valuable model to study sex chromosome turnovers. We characterised X_1_X_2_Y systems in five killifish species and found that sex chromosomes evolved at least four times independently. Sex‐determining regions resided near centromeres or predicted chromosome rearrangement breakpoints in *N. brieni* and *N. guentheri* , suggesting recombination cold spots may facilitate sex chromosome evolution. Chromosomes representing the XY system in *N. furzeri*/*N. kadleci* were sex‐linked also in the outgroup *Fundulosoma thierryi* , with several genes, including *gdf6*, residing in the region of differentiation. Although the X_1_X_2_Y systems of *N. guentheri* , *N. lourensi* (both Coastal clade), and *N. brieni* (Kalahari clade) involved different chromosomes, they shared a potential SD region. We uncovered two sex‐linked evolutionary strata of distinct age in *N. guentheri* . However, its potential SD gene *amhr2* was located in the younger stratum and is hence unlikely to be the ancestral SD gene in this lineage. Our findings suggest recombination landscapes shape sex chromosome turnover and that certain synteny blocks are repeatedly co‐opted as sex chromosomes in killifishes.

## 1 Introduction

The majority of eukaryotes reproduce sexually, and despite the conservation of male and female reproductive functions, mechanisms used to determine sex are remarkably diverse. The most frequent form of sex determination is genotypic, which often involves sex chromosomes. These unique genomic regions represent an intriguing case of evolutionary convergence. Although they evolved repeatedly throughout the Tree of Life by various molecular mechanisms and from different synteny blocks, they share core processes underlying their evolution (Bachtrog et al. 2014). Sex chromosomes mostly evolve from an ordinary autosomal pair through the acquisition of a master sex‐determining (MSD) gene (Charlesworth et al. 2005; Wright et al. 2016; Ponnikas et al. 2018). Recombination is usually arrested around the new sex‐determining (SD) region established on Y or W chromosomes in male or female heterogametic systems, respectively. As the non‐recombining region spreads, the sex‐limited chromosome Y/W degenerates by various mechanisms and eventually becomes cytologically distinct (i.e., heteromorphic) from its counterpart X/Z (reviewed in Kratochvíl et al. 2021). The rate of differentiation is not always correlated with the evolutionary age of a given sex chromosome system (Zhou et al. 2014; Charlesworth 2021; Kuhl et al. 2021). One of the processes that can restart sex chromosome differentiation is sex chromosome turnover. The ancestral sex chromosome system can be replaced by a new one through the evolution of a novel dominant MSD gene or the transposition or translocation of the original MSD to another chromosome. Sex chromosomes can also fuse with autosomes, and this chromosomal addition can start differentiating anew (Pennell et al. 2015; Saunders 2019; Vicoso 2019; Meisel 2020).

Despite over a century of intensive research (reviewed in Abbott et al. 2017), the study of sex chromosome evolution continues to captivate researchers, as many key questions remain unresolved (Saunders and Muyle 2024). Researchers are still exploring the mechanisms driving suppressed recombination between sex chromosomes and why some systems maintain low sequence differentiation, that is, homomorphy, over long evolutionary periods (Charlesworth 2021; Kratochvíl et al. 2021; Smith et al. 2023; Jay et al. 2024). Furthermore, we are only beginning to understand the causes of sex chromosome turnovers and their impact on reproductive isolation and adaptation (Vicoso 2019; Kitano et al. 2024). To address these issues, model systems that feature sex chromosomes in the early stages of differentiation, exhibit variation in sex chromosome systems across species or populations, and possess well‐resolved, time‐calibrated phylogenies, are essential (Charlesworth 2021).

Teleost fishes comprising over half of vertebrate biodiversity (Nelson et al. 2016) meet these criteria. Different sex chromosome systems at various stages of differentiation may be found in closely related species or even in conspecific populations (Kitano and Peichel 2012; Cioffi et al. 2017; El Taher et al. 2021; Sember et al. 2021; Kitano et al. 2024). Besides that, fishes have the highest known diversity of MSD genes among animals, which evolved independently in non‐related species by various molecular mechanisms (Herpin and Schartl 2015; Guiguen et al. 2018; Kitano et al. 2024). Many of these genes, such as *gsdf* (gonadal soma‐derived factor), *gdf6* (growth differentiation factor), *amh* (anti‐Müllerian hormone) or its receptor *amhr2* (Pan et al. 2021; Kitano et al. 2024), belong to the TGF‐ß pathway. The remaining main classes of MSD genes are transcription factors and enzymes involved in the steroidogenic pathway. While reports on novel unusual cases of MSD genes not belonging to the so‐called “usual suspects” have been continually emerging in teleosts in recent years (Kitano et al. 2024; Wang et al. 2024), still they represent a small fraction, suggesting that they are either rare or more difficult to characterise. Thus, there seem to be significant constraints on which genes can become MSD. For instance, *amh* or *amhr2* emerged as MSD in slightly more than half (75/142) documented teleost species according to Wang et al. (2024). For the above reasons, teleosts provide a valuable model for comparative and functional analyses, shedding light on how and why certain genes consistently emerge as key players in the sex‐determining pathway (Herpin and Schartl 2015; Adolfi et al. 2021).

African annual killifishes of the genus *Nothobranchius* Peters, 1868 (Cyprinidontiformes: Nothobranchiidae) occupy an extremely variable environment of ephemeral water bodies in savannahs of southeastern Africa (Wildekamp 2004). They have evolved unique adaptations such as exceptionally short life span with accelerated growth and sexual maturation, and desiccation‐resistant diapausing embryos (Furness 2016; Cellerino et al. 2016; Vrtílek et al. 2018). Given these properties, *Nothobranchius* spp. and particularly the turquoise killifish *N. furzeri* , have become a model for research on ageing (Cellerino et al. 2016; Hu and Brunet 2018). The genus *Nothobranchius* arose ~13 million years ago (MYA) (van der Merwe et al. 2021) and currently comprises more than 90 species (Nagy and Watters 2022; Fricke et al. 2025) distributed across seven main evolutionary clades (van der Merwe et al. 2021). All species typically live in small, geographically isolated populations. The resulting genetic drift has considerably influenced the evolution of their genomes and diversification (Bartáková et al. 2013; Cui et al. 2019; van der Merwe et al. 2021). Available data suggest relatively recent diversification within the clades (Dorn et al. 2014; van der Merwe et al. 2021), implying a fast rate of ongoing allopatric speciation (Bartáková et al. 2013; Dorn et al. 2014).

*Nothobranchius* species demonstrate dynamic genome evolution via frequent chromosomal rearrangements (Krysanov and Demidova 2018; Krysanov et al. 2016, 2023), resulting in a notable diversity of diploid chromosome numbers (2n = 16–50) (Krysanov and Demidova 2018) and shifts in repeat content (Reichwald et al. 2009; Cui et al. 2019; Štundlová et al. 2022; Voleníková et al. 2023). Two sister species, *N. furzeri* and *N. kadleci*, share the ♀XX/♂XY sex chromosome system (Reichwald et al. 2015; Štundlová et al. 2022) with the Y‐linked allele of the *gdf6* gene (*gdf6Y*) identified thus far as the only MSD gene in *Nothobranchius* spp. (Reichwald et al. 2015; Štundlová et al. 2022; Richter et al. 2025). Six other representatives possess multiple sex chromosome systems of the ♀X_1_X_1_X_2_X_2_/♂X_1_X_2_Y type, which was also reported in the outgroup species *Fundulosoma thierryi* (Ewulonu et al. 1985; Krysanov et al. 2016; Krysanov and Demidova 2018; Simanovsky et al. 2019). Cytogenetic investigations suggest that this system has most probably resulted from Y‐autosome fusion; hence X_1_ denotes the original X chromosome and X_2_ corresponds to a chromosome which has fused to the Y chromosome. Available data is not sufficient to distinguish whether these multiple sex chromosome systems have a single origin or evolved independently. The latter is, however, favoured (Krysanov and Demidova 2018; Simanovsky et al. 2019) as sex chromosome morphology differs among species in question, namely *N. brieni* , *N. ditte*, *N. guentheri* , *N. janpapi* , *N. lourensi* and *Nothobranchius* sp. Kasenga, which are scattered across the *Nothobranchius* phylogeny, nested within species with unknown sex chromosome constitution (Krysanov and Demidova 2018; van der Merwe et al. 2021; Bartáková et al. 2025).

Differentiation of X and Y chromosomes varies greatly between populations of *N. furzeri* and *N. kadleci* due to large inversions (Reichwald et al. 2015; Štundlová et al. 2022). The degree of differentiation of the *Nothobranchius* X_1_X_2_Y sex chromosomes has been so far assessed only indirectly, based on the distribution of constitutive heterochromatin and hybridisation patterns of repetitive sequences (Lukšíková et al. 2023; Voleníková et al. 2023). With the sole exception of *N. brieni* , these analyses showed a generally low degree of accumulation of heterochromatin and repetitive sequences along with minor differences between gametologs, corroborating the usual pattern found in the majority of fish multiple sex chromosomes (Sember et al. 2021).

In the present study, we combined molecular cytogenetic and genomic methods to determine whether *Nothobranchius* X_1_X_2_Y sex chromosome systems had recurrent and independent origins, and to assess levels of their differentiation and identify their putative MSD genes. We revealed at least four sex chromosome turnovers in *Nothobranchius* killifishes and *F. thierryi* . The Y‐linked non‐recombining regions of *F. thierryi* and *N. guentheri* contained *gdf6* and *amhr2* of the TGF‐ß pathway, respectively. A possible role of these “usual suspects” in sex determination of these species is discussed.

## 2 Material and Methods

### 2.1 Fish Species Sampling

We analysed four *Nothobranchius* species with an X_1_X_2_Y sex chromosome system, namely *N. brieni* , *N. ditte*, *N. guentheri* and *N. lourensi* , and the outgroup species *Fundulosoma thierryi* carrying the same type of multiple sex chromosomes. To compare the results to our earlier findings (Štundlová et al. 2022), we also included *N. furzeri* with the XY sex chromosome system. A single population per species has been sampled except for *N. guentheri* (two populations). The studied individuals from *N. furzeri* were sampled from the laboratory population (see Bartáková et al. 2015; Blažek et al. 2017). The remaining species were obtained from specialists and experienced hobby breeders who keep strictly population‐specific lineages derived from original imports. In this case, the species identity was confirmed based on key morphological characters (Wildekamp 1996, 2004; Nagy 2018). The sampling summarised in Table 1 is relevant to cytogenetic methods, while samples for genomic approaches are specified in the respective sections.

**TABLE 1:** List of *Nothobranchius* killifish species used in this study, with assignment to their phylogeographic lineage.

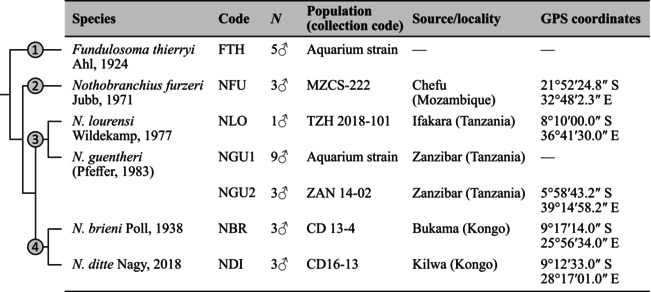

*Note:* Numbered lineages correspond to (1) outgroup, (2) Southern, (3) Coastal and (4) Kalahari clade, population/collection codes, sample sizes (*N*) for cytogenetic analyses, source/geographic origin and GPS coordinates of sampling localities. Phylogenetic relationships follow van der Merwe et al. (2021) and the exact placement of *N. lourensi* in the phylogeny is based on recent phylogenomic analysis of Tanzanian *Nothobranchius* species using double‐digest restriction site‐associated DNA (ddRAD) sequencing (Bartáková et al. 2025). Additional information to the origin of the sampled individuals is provided in the main text, section “Fish Species Sampling”. Except for *N. lourensi* , at least three male individuals per species have been studied per each cytogenetic method.

### 2.2 DNA Isolation

For molecular cytogenetic experiments and short‐read genome sequencing, high molecular weight genomic DNA (HMW gDNA) from male individuals of all species under study and *F. thierryi* and *N. guentheri* females was extracted from liver, spleen, and brain tissue using a MagAttract HMW DNA Kit (Qiagen, Hilden, Germany) following the manufacturer's instructions, doubling the volume of all reagents and buffers except for the elution buffer. HMW gDNA for long‐read sequencing was extracted using the Nanobind tissue kit (PacBio, Menlo Park, CA, USA) following the application note for isolation of HMW DNA from frozen tilapia testis tissue. The remaining samples, particularly for the PCR assay in *N. guentheri* (see below), were isolated using the Tissue DNA Isolation Kit (Geneaid Biotech, Taipei, Taiwan).

### 2.3 Read Control Quality and Genome Size Estimation Using GenomeScope

To assemble their genome sequence, we sequenced *F. thierryi* female genome and *N. guentheri* male genome using ONT (Oxford Nanopore Technologies, Oxford, UK) and PacBio (Pacific Biosciences, Menlo Park, CA, USA) technology, respectively. We also re‐sequenced male and female gDNA in both species using the Illumina NovaSeq 6000 platform and an S4 flow‐cell (Illumina, San Diego, CA, USA) to correct the error‐prone long reads and perform coverage analyses. Illumina sequencing was performed by Novogene (Hong Kong, China). Library preparation and ONT sequencing on the PromethION were also performed by Novogene. Library preparation and PacBio CLR sequencing were performed by the Genomics & Cell Characterisation Core Facility of the University of Oregon (Oregon, USA) using the PacBio Sequel II system and one 8M SMRT cell.

The raw Illumina sequencing data underwent quality assessment using FastQC (Andrews 2010) and were subsequently trimmed with Trimmomatic v0.39 (Bolger et al. 2014). Oxford Nanopore Technology and PacBio reads were quality‐checked using the NanoPack tool (De Coster et al. 2018), specifically utilising NanoPlot for assessment and NanoFilt for filtering and trimming. Based on the visualised quality of the sequencing, ONT reads under 10 kb in length and with a quality score below nine were excluded from the dataset. The Ratatosk tool (Holley et al. 2021) was employed with default settings to correct ONT reads using highly accurate Illumina reads from the same sample. As for PacBio data, only reads longer than 5 kb were used for genome assembly.

For estimating genome sizes, GenomeScope2 (Ranallo‐Benavidez et al. 2020) was used with the Jellyfish k‐mer counting tool (Marçais and Kingsford 2011). Various k‐mer lengths ranging from 15 to 45 were used, with *k* = 45 ultimately selected for its optimal model fit value for *F. thierryi* and 21 for *N. guentheri* .

### 2.4 Genome De Novo Assembly and Its Quality Evaluation

The *F. thierryi* female Nanopore long reads were first corrected using the Illumina short reads. Then, several options were used for genome assembly using Flye v2.8 (Kolmogorov et al. 2019) with the *“—nano‐raw”* and “*‐‐min‐overlap 5000*” option giving the best assembly in terms of contiguity and BUSCO (Benchmarking Universal Single‐Copy Orthologs) completeness. Moreover, an appropriate genome size estimated by GenomeScope2 was also set in the assembly process. The assembly underwent one round of long read polishing via medaka (https://github.com/nanoporetech/medaka) followed by a round of short read polishing using NextPolish (Hu et al. 2020) with Illumina reads. Haplotypic duplicates were then removed using the purge_dups v1.0.1 tool (Guan et al. 2020). Contamination checks were performed using BlobTools v1.0 (Laetsch and Blaxter 2017), and any contigs associated with non‐target organisms were eliminated using the “*seqfilter*” function. The best result for *N. guentheri* male PacBio genome assembly was achieved using Flye v2.8 (Kolmogorov et al. 2019) with the “—pacbio‐raw” option, genome size not defined, and without further polishing or de‐duplication. The assemblies were quality checked with BUSCO v5 with the Actinopterygii database (odb_10) (Manni et al. 2021).

### 2.5 Genome Annotation

Functional and structural annotations were conducted using the GenSAS v6.0 pipeline (Humann et al. 2019). Repetitive sequences were identified employing RepeatModeler2 (Flynn et al. 2020) utilising the RMBlast search engine and modules including TFR v4.09, RECON, and RepeatScout v1.0.5. Additionally, TAREAN (Novák et al. 2017) was utilised for satDNA annotation. All consensus sequences marked as satellites by TAREAN, regardless of confidence levels, were integrated into a custom database as dimers to enhance the satellite DNA annotation. RepeatMasker v4.1.1 (Smit et al. 2013‐2015, available at http://www.repeatmasker.org) with the NCBI/RMBlast search engine was employed for repeat annotation. This involved a combination of the newly identified repeats from RepeatModeler2 and the custom database containing the satDNA sequences from TAREAN.

RNA‐seq data were used for *N. guentheri* male genome annotation. To that end, total RNA was extracted from brain tissue dissected from *N. guentheri* . Subsequently, the 150 bp Illumina reads were aligned to the respective genomes using STAR v2.7.7 (Dobin and Gingeras 2015). The genome index required for mapping was generated using the following command: *STAR ‐‐runThreadN 9 ‐‐runMode genomeGenerate ‐‐genomeDir*/*genomedir ‐‐genomeFastaFiles*<*genome>‐‐genomeSAindexNbases 13*. Following index generation, mapping was executed using the command: *STAR ‐‐runThreadN 9 ‐‐genomeDir/genomedir ‐‐readFilesIn/Forward.fq* /*Reverse.fq*. The resulting SAM file was converted to BAM format using the SAMtools suite (v1.11) (Li et al. 2009). The generated BAM file was then utilised for gene prediction through BRAKER2 with default settings, which incorporates Augustus and GeneMark‐EP (Lomsadze et al. 2014; Brůna et al. 2021). For the annotation of *F. thierryi* female genome assembly, Augustus v3.3.1 (Stanke and Morgenstern 2005) and GeneMark‐ES were used directly, without RNA‐seq evidence to guide the process. Furthermore, tRNA and rRNA sequences were identified in all assemblies using tRNAscan‐SE v2.0.7 (Chan and Lowe 2019) and RNAmmer v1.2 (Lagesen et al. 2007), respectively.

### 2.6 Reference Guided Scaffolding

To obtain pseudochromosome level assemblies of both, *F. thierryi* and *N. guentheri* , we aligned genome assembly contigs to chromosomes of the *N. furzeri* reference (GenBank acc. no. GCA_014300015.1; Willemsen et al. 2020) using Minimap2 v2.17 (Li 2018) and sorted and oriented the contigs into pseudomolecules using RaGOO (Alonge et al. 2019) with “*‐b ‐s*” options.

### 2.7 Pool‐Seq Analysis

To identify male‐specific (MSY) regions, we generated pooled samples of genomic DNA from *N. guentheri* (28 males, 29 females) and *F. thierryi* (20 males, 11 females) and sequenced them in paired‐end mode on the NovaSeq 6000 Illumina platform with Novogene. The Illumina pools were quality checked with FastQC v0.11.5 (Andrews 2010) and filtered with the “*‐‐nextseq‐trim = 20 ‐‐minimum‐length = 100*” options using cutadapt v1.15 (Martin 2011) and trimmed with Trimmomatic v0.36 (Bolger et al. 2014) with following parameters: “*SLIDINGWINDOW:4:25 MINLEN:100 HEADCROP:4 CROP:140*”. The trimmed and filtered reads from female and male pools were mapped separately to the corresponding RaGOO pseudochromosomes using BWA‐MEM v0.7.17 (Li and Durbin 2009) with default parameters. Using Picard Toolkit (https://broadinstitute.github.io/picard/) we sorted resulting BAM files by coordinate (“*SortSam SORT_ORDER = coordinate*”) and removed PCR duplicates (“*MarkDuplicates REMOVE_DUPLICATES=true REMOVE_SEQUENCING_DUPLICATES=true*”). Subsequently, we generated a file with the nucleotide composition of all genomic positions using the *pileup* function of the software Pooled Sequencing Analysis for Sex Signal (PSASS; Feron and Jaron 2021). We used PSASS to identify non‐overlapping 50 kb windows enriched in sex‐specific single‐nucleotide polymorphisms (SNPs), using the following parameters: “*‐‐min‐depth 10, ‐‐freq‐het 0.5 ‐‐range‐het 0.15 ‐‐freq‐hom 1 ‐‐range‐hom 0.05 ‐‐window‐size 50000 ‐‐output‐resolution 50000 ‐‐group‐snps*”.

### 2.8 Coverage Analysis

The short Illumina reads from three male and female samples of both *F. thierryi* and *N. guentheri* were quality checked with FastQC v0.11.5 (Andrews 2010) and filtered with “*‐‐nextseq‐trim = 20 ‐‐minimum‐length = 100*” options using cutadapt v1.15 (Martin 2011) and trimmed with Trimmomatic v0.36 (Bolger et al. 2014) with following parameters: “*SLIDINGWINDOW:4:25 MINLEN:100 HEADCROP:10 CROP:130*”. Repetitive sequences of pseudochromosome‐level assemblies were identified by RepeatModeler v1.0.11 (Smit and Hubley 2008‐2015, available at http://www.repeatmasker.org) and annotated by RepeatMasker v4.0.7 (Smit et al. 2013‐2015, available at http://www.repeatmasker.org) with the NCBI search. Filtered reads were mapped to the corresponding masked reference genome via Bowtie2 v2.2.9 (Langmead and Salzberg 2012) with the “*‐‐very‐sensitive‐local ‐‐no‐discordant ‐‐no‐mixed*” parameters and the outputs were compressed to BAM format using SAMtools *view* (v1.3.1; Li et al. 2009) and merged according to sex using SAMtools *merge*. The resulting BAM files were then parsed using utilities from the Bedtools suite v2.25.0 (Quinlan and Hall 2010). A genome file was parsed from the BAM files using SAMtools *view* and divided into 50 kbp sliding windows using Bedtools *makewindows* with “*‐w 50000 –s 50000*” parameters. The merged BAM files were sorted with SAMtools *sort* and converted to BED format using Bedtools *bamtobed* “*‐split*”. Finally, the per base coverage of aligned sequences within 50 kbp windows spanning the genome was computed using Bedtools *coverage*. We excluded reads which mapped to regions corresponding to repetitive sequences identified by RepeatModeler using the Bedtools *subtract* command. Finally, we obtained median coverage depth for each 50 kbp window using Bedtools *genomecov*. In both sexes, coverage depths for each scaffold were normalised by mean coverage across scaffolds and compared between sexes, formulated as the Log2 of the male:female (M:F) coverage ratio. The resulting data, together with those from Pool‐seq analysis, were visualised using “*SexGenomicsToolkit/sgt*r” R package (https://github.com/SexGenomicsToolkit/sgtr). Autosomal and pseudoautosomal regions should present a Log2(M:F) = 0 as they are expected to be present in equal proportion in both sexes, while the fully differentiated X‐linked scaffolds should ideally present a Log2(M:F) = −1 due to its double representation in females. Sequences enriched or specific to Y chromosome produce large positive Log2(M:F) values. Log2(M:F) coverage values can exceed the expectations due to undetected repeats amplified on X or Y chromosomes.

### 2.9 Delimitation of Evolutionary Strata on *F. thierryi* and *N. guentheri* Sex Chromosomes

To determine boundaries of evolutionary strata with distinct levels of differentiation, we applied changepoint analysis to detect variance shifts in density of male‐specific SNPs, F_ST_ and Log2(M:F) coverage values. Analyses were performed using the “*cpt.var*” function from the “*changepoint*” R package (Killick and Eckley 2014). For *F. thierryi* Chr14 and Chr3, we applied the “*PELT*” method for multiple changepoint detection using a manual penalty value “*pen.value = 20*” and a minimum segment length “*minseglen = 60*”. For *N. guentheri* , we used the “*PELT*” method for Chr13 and “*AMOC*” (*method = “AMOC”*) method for a single changepoint detection for Chr11, both with the Schwarz Information Criterion (*penalty = “SIC”*). After overlaying the changepoints from all three statistics, we visually inspected the combined plots and retained the breakpoint that best captured a common step‐change across all statistics. These selected changepoints were then taken as the putative boundaries of the evolutionary strata along each chromosome.

### 2.10 Search for Common Sex‐Determinants in *F. thierryi* and *N. guentheri* Genomes

Genes that are commonly associated with sex determining function among fishes as well as other sex‐related genes were searched in the *F. thierryi* and *N. guentheri* genomes assembled in this study (Table S1), both primary Flye assemblies and RaGOO pseudochromosomes. We employed tBLASTx search to identify common sex determining loci in the *F. thierryi* and *N. guentheri* genomes (Pan et al. 2021; Kitano et al. 2024). Accession numbers of sequences used as queries are listed in Table S1. Searches using tBLASTx algorithm (Camacho et al. 2009) were used with “*‐max_target_seqs 3 ‐evalue 1e‐25 ‐outfmt 6*” options.

### 2.11 Variant Detection

To analyse allelic variants in the putative SD genes, namely *hsd17b* and *gdf6* in *F. thierryi* and *amhr2* in *N. guentheri* , we mapped paired‐end short read Illumina Pool‐seq data to pseudochromosomes of respective species scaffolded by RaGOO. Male and female Pool‐seq data were trimmed and filtered using TrimGalore v0.6.2 (Krueger et al. 2023) using the following flags: *“‐‐fastqc ‐‐clip_R1 10 ‐‐clip_R2 10 ‐‐three_prime_clip_R1 10 ‐‐three_prime_clip_R2 10 ‐‐paired*”. Effectivity of the trimming step was verified using FastQC v0.11.9 (Andrews 2010). Trimmed reads were then mapped using Bowtie2 v2.3.5.1 (Langmead and Salzberg 2012) with *“‐‐very‐sensitive‐local ‐‐no‐mixed ‐‐no‐discordant*” flags. Variants were called using the resulting alignment files as an input for Freebayes v9.9.2 (Garrison and Marth 2012) in default mode. By visualising the alignment files in Geneious Prime v2023.2.1, we obtained the compositions of the SNPs for males and females. The Y‐linked SNPs were identified based on manual inspection of the Pool‐seq data alignment stacks in a target region. Only well represented variants heterozygous in males and missing in females were declared as Y‐linked. The T>C substitution in the exon 5 of the *N. guentheri* *amhr2* Y‐linked allele was verified by a duplex PCR assay using two sets of primers, each amplifying different regions of the *amhr2* alleles: NguAMHR_F1 (5′‐TTGGCCTGAAGAACACATCTACA‐3′) with NguAMHR_R1 (5′‐ACAACGGGCTGTGAGGATAG‐3′) amplifying the segment common to both X and Y *amhr2* alleles (expected fragment size: 156 bp), and NguAMHR_F2 (5′‐ACCTGCTGCTATCACTCCAG‐3′) with NguAMHRY_R2 (5′‐TCATTAATTTAAAACCAGCCTGACTA‐3′) specific for Y‐chromosomal allele as it contained the male‐specific SNP at NguAMHRY_R2 3′‐end (expected fragment size: 411 bp). Primers were synthesised by Generi Biotech (Hradec Králové, Czech Republic). We tested 15 males and 11 females from the population NGU1, and four males from NGU2. The PCR reaction (10 μL in total) consisted of 1× Multiplex PCR Master Mix (BiotechRabbit Gmbh, Berlin, Germany), 500 nM of each primer and 30–50 ng of gDNA. Thermocycling conditions were as follows: one cycle with denaturation for 3 min at 95°C, followed by 29 cycles of 30 s at 95°C, 30 s at 63°C and 30 s at 72°C, with the final primer extension prolonged to 3 min at 72°C. For additional verification by Sanger sequencing (SEQme, Dobříš, Czech Republic), we used primer NguAMHR_F2 in combination with NguAMHR_R3 (5′‐GGTGTGAAGATGGTAGCTCC‐3′).

### 2.12 Haplotype‐Specific Assembly of Chromosome 13

To separately assemble the male and female haplotypes of the putative sex determining region on the *N. guentheri* chromosome 13 (NguChr13), we have reassembled this chromosome using the trio binning mode. Original PacBio long reads used for the *N. guentheri* reference genome assembly were mapped back to it using Minimap2 v2.22 (Li 2018) with the flags “*‐a ‐x map‐pb*”. From the resulting alignment file, only reads mapping in the Chr13 were selected using Samtools v1.11 (Danecek et al. 2021). Subsequent conversion of alignment file into FASTQ format was done using Bedtools bamtofastq with default setting (Quinlan and Hall 2010). Resulting reads were used as a sequence set for the new NguChr13 assembly performed using Canu v2.2 (Koren et al. 2017) with the following flags enabled “*genomeSize=30m ‐‐pacbio [filename] ‐haplotypePAT [patname] ‐haplotypeMAT [matname]*”. Parental Illumina short reads were used to enable the binning. Assembly process resulted in the creation of two Chr13 haplotypes, one maternal and the other paternal.

### 2.13 Conventional Cytogenetics

Mitotic chromosome spreads were obtained either from regenerating caudal fin tissue or from cephalic kidney. In the former, we followed Völker and Ráb (2015) PRJNA1286812, with the modifications by Sember et al. (2015), and with an altered time of fin regeneration (1 to 2 weeks; in *N. lourensi* up to 3 weeks). For the kidney‐derived chromosome preparations, and for the preparation of meiotic spreads from gonads in some individuals, we followed either Ráb and Roth (1988) or Kligerman and Bloom (1977). The latter protocol was modified according to Krysanov and Demidova (2018). The chromosomal spreading quality was enhanced following Bertollo et al. (2015). Preparations were inspected under phase‐contrast for the amount and quality of chromosome spreads. Suitable slides were dehydrated in an ethanol series (70%, 80% and 96%, 2 min each) and stored at −20°C until further use.

### 2.14 Whole Chromosome Painting (WCP) and Zoo‐FISH (Cross‐Species Fluorescence in Situ Hybridization) With Sex Chromosome‐Specific Probes

To assess the degree of shared synteny between multiple sex chromosomes and to verify the mechanism of their origin, we prepared three WCP probes generated from multiple sex chromosomes of different *Nothobranchius* species. In the case of *N. guentheri* and *N. lourensi*, we cut out easily discernible large Y chromosomes from mitotic metaphases (10 and 8 copies, respectively). In the case of *F. thierryi* , we did not have suitable mitotic slides for this procedure; therefore, we isolated a whole sex‐trivalent from meiotic spreads (8 copies), based on its characteristic morphology (see Figure S1A).

The glass needle‐based microdissection was carried out under an inverted microscope (Zeiss Axiovert 135, Zeiss, Oberkochen, Germany) using a sterile glass needle attached to a mechanical micromanipulator (Zeiss) (a procedure detailed in Al‐Rikabi et al. 2020). The primary chromosome material was amplified and labelled (by SpectrumOrange‐dUTP or SpectrumGreen‐dUTP; both Vysis Downers Grove, IL, USA) in two subsequent reactions of degenerate oligonucleotide‐primed (DOP) PCR following Yang et al. (2009). The resulting painting probes were designated as NGU‐Y (Y chromosome of *N. guentheri* ), NLO‐Y (Y chromosome of *N. lourensi* ), and FTH‐triv (entire sex trivalent of *F. thierryi* ). The final probe cocktail for each slide was composed of two compared WCP probes (100 ng each) supplemented with 9 μg of unlabelled blocking DNA, to block non‐specific hybridisation by highly repeated DNA sequences (4.5 μg DNA of an analysed species and the remaining 4.5 μg comprising equal amount of gDNA of species from which the WCP probes have been generated). Zoo‐FISH procedure was performed using a combination of two previously published protocols. Specifically, the slide pre‐treatment, probe/chromosome denaturation and hybridisation followed Sember et al. (2015), while post‐hybridisation washing was done according to Yano et al. (2017) with slight modifications described in Štundlová et al. (2022). After standard post‐hybridisation washes, the slides were passed through an ethanol series and mounted in antifade containing 1.5 μg/mL DAPI (4′,6‐diamidino‐2‐phenylindole; Cambio, Cambridge, United Kingdom).

### 2.15 Fluorescence in Situ Hybridization (FISH) With 5S and 18S rDNA Probes

Our previous results from Chromomycin A_3_ staining (Voleníková et al. 2023) suggested that the additional WCP signals (see Results section and Figure S1C,D) could colocalise with rRNA gene clusters (rDNA). To test it, we used FISH with 5S and 18S rDNA fragments. For the probe preparation, we used previously cloned PCR fragments of 18S rDNA from *N. guentheri* and 5S rDNA from *N. kadleci*, both successfully used in our previous study (Štundlová et al. 2022). The probe composition and FISH conditions followed Sember et al. (2015) with slight modifications specified in Štundlová et al. (2022).

### 2.16 Bacterial Artificial Chromosome (BAC) Clone Isolation, Sequencing, and Nick‐Translation

We mapped *N. furzeri* BAC clones (Table 2) carrying genes commonly involved in fish sex determination (Table S1) onto chromosomes of the studied species. The BAC clones were selected by BLASTn search using *N. furzeri* BAC end sequences and genome sequence (Reichwald et al. 2015), selecting those clones for which both tags mapped within a 100–150 kb window around candidate genes (Table 2). BAC DNA was isolated using the NucleoBond Xtra Midi kit (Macherey Nagel, Düren, Germany) and verified by PCR with specific primers (Table S2). Selected BAC clones were further sequenced using the SQK‐LSK109 Ligation Sequencing Kit (Oxford Nanopore Technologies, Oxford, UK) along with other BAC clones (not part of this study) on the MinION mk1b platform using the R9.4.1 flow cell (Oxford Nanopore Technologies) and basecalled, filtered, and assembled following Pospíšilová et al. (2023) with slight modifications. The obtained data in fast5 format were basecalled with guppy software version 6.0.6 (Oxford Nanopore Technologies) using a high‐precision flip‐flop algorithm. The resulting reads were filtered for quality (Q > 10) and length (> 10 kbp) using NanoFilt (De Coster et al. 2018). The filtered reads were assembled using Flye software version 2.9 (Kolmogorov et al. 2020) with the “—*meta*”parameter except that the minimal overlap was set up to 2000. Contigs containing genes of interest were identified using BLAST+ version 2.12.0 (Camacho et al. 2009) with the blastn algorithm, selecting only contigs with > 99% match. The ends of BAC clones and genes were mapped to the selected contigs using minimap2 version 2.17 (Li 2018) with a parameter for mapping high‐quality spliced alignments. Geneious Prime 2022.2.2 was used for annotation of genes and visualisation of circular BAC clones.

**TABLE 2:** BAC clones from *N. furzeri* genome library which carry specific genes (following information from supplement S1I in Reichwald et al. 2015) used in fluorescence in situ hybridization.

BAC clone	Gene	*F. thierryi*	*N. guentheri*	*N. lourensi*	*N. brieni*	*N. ditte*
GRZ‐B‐a‐201Bg09	*amh*		X			X
GRZ‐B‐a‐156Cc04	*amhr2*	X	X	X	X	X
GRZ‐B‐a‐8Dd06	*bcar1*					X
GRZ‐B‐a‐178 Ah11	*DMRT3*		X			X
GRZ‐B‐a‐8Bg10	*gdf6*			X		
GRZ‐B‐a‐201 Bd03	*gdf6*	X	X	X	X	X
GRZ‐B‐a‐243Ch05	*gsdf*					X
GRZ‐B‐a‐242Cg02	*hsd17b*		X			X
GRZ‐B‐a‐170Bg04	*SOX3*		X			X

*Note:* BAC clones used in screening of particular species are marked as “X”.

Labelling of isolated BAC DNA was done by the nick‐translation method. The reaction contained 2 μg of BAC DNA, 1× nick translation buffer (5 mM Tris–HCl, 0.5 mM MgCl_2_, 0.0005% bovine serum albumin, BSA; pH 7.5), 10 mM β‐mercaptoethanol, 50 μM dATP, 50 μM dCTP, 50 μM dGTP, 10 μM dTTP, 20 μM Cy3‐dUTP or Fluorescein‐12‐dUTP, 40 U DNA polymerase I (Thermo Fisher Scientific, Waltham, MA, USA) and 0.01 U DNase I (RNase‐free, Thermo Fisher Scientific). The reaction was incubated at 15°C for 5 h and finally stopped by adding a 1× loading buffer (50% glycerol, 250 mM EDTA, 5.9 mM bromphenol blue).

### 2.17 BAC‐FISH

The FISH experiments were performed using a combination of three previously published protocols (Sahara et al. 1999; Sember et al. 2015; Yano et al. 2017), with slight modifications described in Štundlová et al. (2022). Briefly, the hybridization mixture was composed of 300 ng of Cy3‐labelled BAC DNA and 500 ng of FITC‐labelled BAC DNA, 6 μg of thermally fragmented male competitive gDNA (99°C, 20 min), 1500 ng of DOP‐PCR‐derived competitive DNA from male gDNA (based on Yang et al. 2009) and 25 μg of sonicated salmon sperm DNA (Sigma‐Aldrich, St. Louis, MO, USA). The hybridization mixture was denatured for 5 min at 90°C. After application of the probe cocktail on the slide, the hybridization took place in a moist chamber at 37°C for 3 days. Subsequently, non‐specific hybridization was removed by post‐hybridization washes. Specifically, slides were treated once 5 min at 62°C in 1% Triton X‐100 in 0.1× SSC and then 2 min at room temperature (RT) in 1% Triton X‐100 in 2× SSC. Final wash 2 min at RT in 1% Kodak PhotoFlo in H_2_O was followed by counterstaining with 0.2 g/mL DAPI mounted in antifade based on DABCO (1,4‐diazabicyclo(2.2.2)‐octane; Sigma‐Aldrich). Slides reused for repeated FISH experiments were reprobed following Yoshido et al. (2015).

In some instances, WCP and BAC‐FISH probes were successfully mapped simultaneously in a combined experiment, following the WCP protocol, as described above.

### 2.18 Microscopic Analyses and Image Processing

Images from the majority of cytogenetic experiments were captured under immersion objective 100× using BX53 Olympus microscope (Olympus, Tokyo, Japan) equipped with an appropriate fluorescence filter set, and coupled with a black and white CCD camera (DP30W Olympus). Images were acquired for each fluorescent dye separately using DP Manager imaging software (Olympus). The same software was used to superimpose the digital images with the pseudocolours (blue for DAPI, red for Cy3 and SpectrumOrange, green for FITC or SpectrumGreen). Composite images were optimised and arranged using Adobe Photoshop, version CS6 (Adobe Systems, San Jose, CA, USA). Images from BAC‐FISH and most of the combined WCP/BAC‐FISH experiments were captured using Leica DM6 B Fluorescence Microscope (Leica Microsystems, Wetzlar, Germany) equipped with Leica sCMOS Monochrome Camera DFC9000GT, using the Leica Application Suite X (LAS X) imaging software v3.7.3.23245.

At least 20 chromosome spreads per individual in each experiment were analysed; some of them were reprobed several times. The description of chromosome morphology, based on Levan et al. (1964), was modified as: m—metacentric, sm—submetacentric (biarmed chromosomes), st—subtelocentric, and a—acrocentric (monoarmed chromosomes).

To compare chromosomal homeologies and the localisation of the *gdf6* and *amhr2* genes, we measured chromosomes from three representative mitotic metaphases per species using ImageJ with the Levan plugin (Sakamoto and Zacaro 2009). Our objectives were to: (i) determine the relative chromosome length as a percentage of the total length of diploid karyotype and the chromosome arm ratio for each chromosome, (ii) measure the relative length of homeologous blocks painted by WCP and their relative distance from chromosome ends, and (iii) determine the positions of the *gdf6* and *amhr2* genes. Due to significant variation in the centromere size, particularly in *N. furzeri* , the relative position of the markers was measured separately on both chromosome arms, with the zero point always set at the tip, extending to the centromere. The extent of the WCP‐labelled segment spanning the entire chromosome or chromosome arm was not measured.

## 3 Results

### 3.1 Genome Assemblies and Annotation

The raw read counts and lengths for genome sequencing samples, coverage and other statistics are detailed in Tables S3–S5. A summary of genome assembly statistics for both species is outlined in Table 3. The assemblies exhibited contig N50 values of 6 Mbp and 15 Mbp for *F. thierryi* female and *N. guentheri* male, respectively. While BUSCO analyses showed the assemblies to be on par with the *N. furzeri* reference genome (GCA_014300015.1, Willemsen et al. 2020) with about 89% of conserved orthologs to be complete in both species under study (Table 3). Both assemblies thus served as high‐quality references suitable for downstream analyses. However, the gene annotation was admittedly poor probably due to lack of RNA‐seq data and *N. furzeri* gene models were used to delimit intron‐exon boundaries where needed (see below). Genome assemblies and annotation files were deposited in the Dryad repository (Hospodářská et al. 2024).

**TABLE 3:** Summary statistics for *Fundulosoma thierryi* female and *Nothobranchius guentheri* male genome assemblies.

Assembly stats	*F. thierryi*	*N. guentheri*	*N. furzeri* *
Total length (Mbp)	983	940	986.5
N fragments	1621	1021	40,229
N50 (kbp)	6062.3	15,275.3	49,900.0
Largest contig (Mbp)	28.078	54.174	74.218
BUSCO (C; D; F; M) (%)	88.9; 1.3; 2; 9.1	89.2; 1.4; 1.9; 8.9	87.7;1.9; 1.8; 10.5
*N* of genes	42,757	107,896	20,242

Abbreviations: BUSCO, Benchmarking Universal Single‐Copy Orthologs; C, complete; D, duplicated; F, fragmented; M, missing.

* GenBank acc. no. GCA_014300015.1.

### 3.2 Identification of Sex‐Linked Regions in *F. thierryi* and *N. guentheri*

**FIGURE 1:**
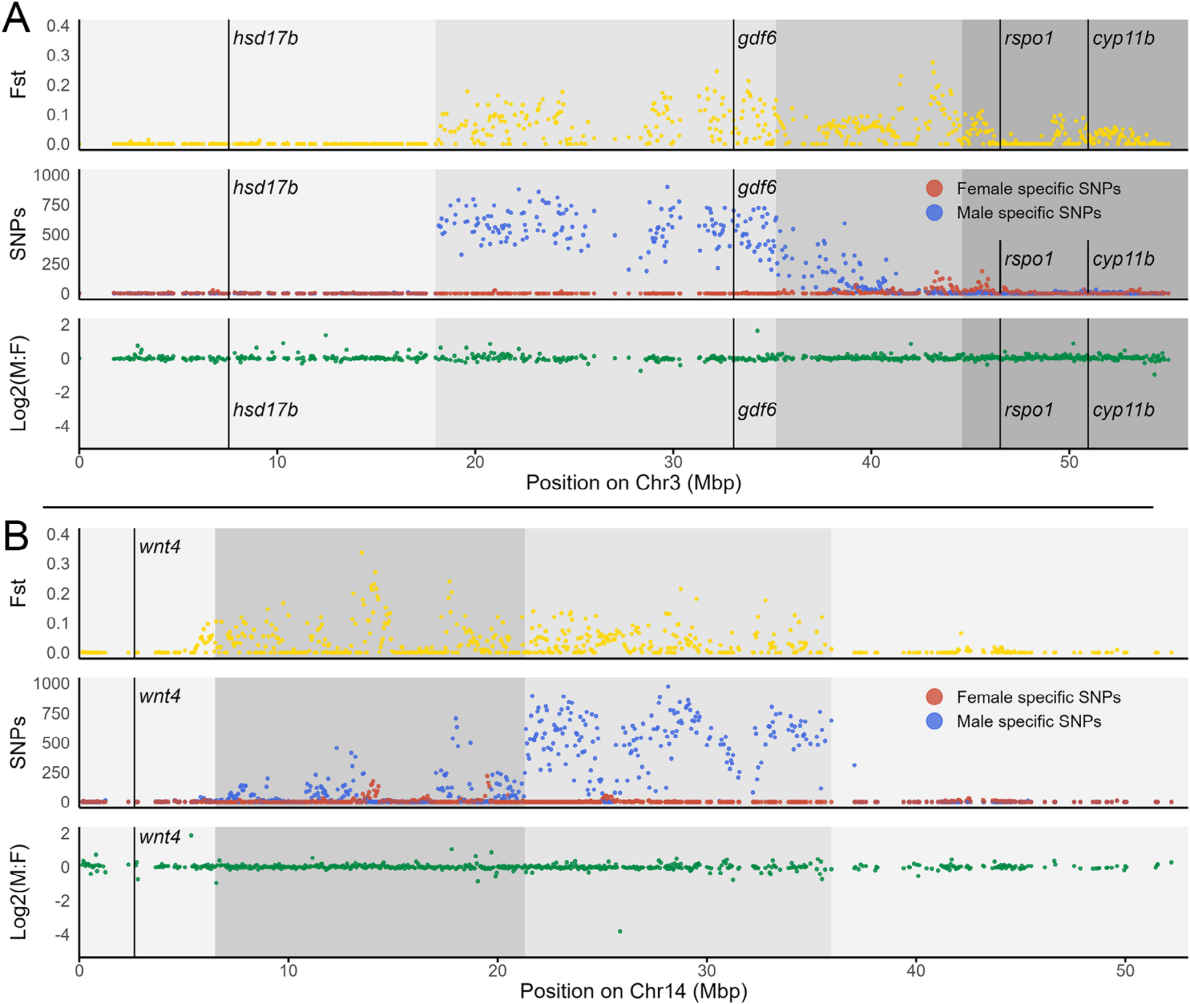
Sex‐linked regions in *F. thierryi* . Plots show results of Pool‐seq analyses [male and female specific SNPs and relative divergence (F_ST_) are in blue, red and yellow, respectively] and coverage (in green) using pseudochromosomes constructed with *N. furzeri* as a reference (Willemsen et al. 2020). (A) chromosome 3 corresponds to the *N. furzeri* sex chromosome; (B) chromosome 14. Grey shading marks putative boundaries of the evolutionary strata along each chromosome. Note that these chromosomes correspond to X_1_ and X_2_ chromosomes, which pair with the neo‐Y chromosome of fusion origin. Results for all chromosomes are shown in Figure S2.

**FIGURE 2:**
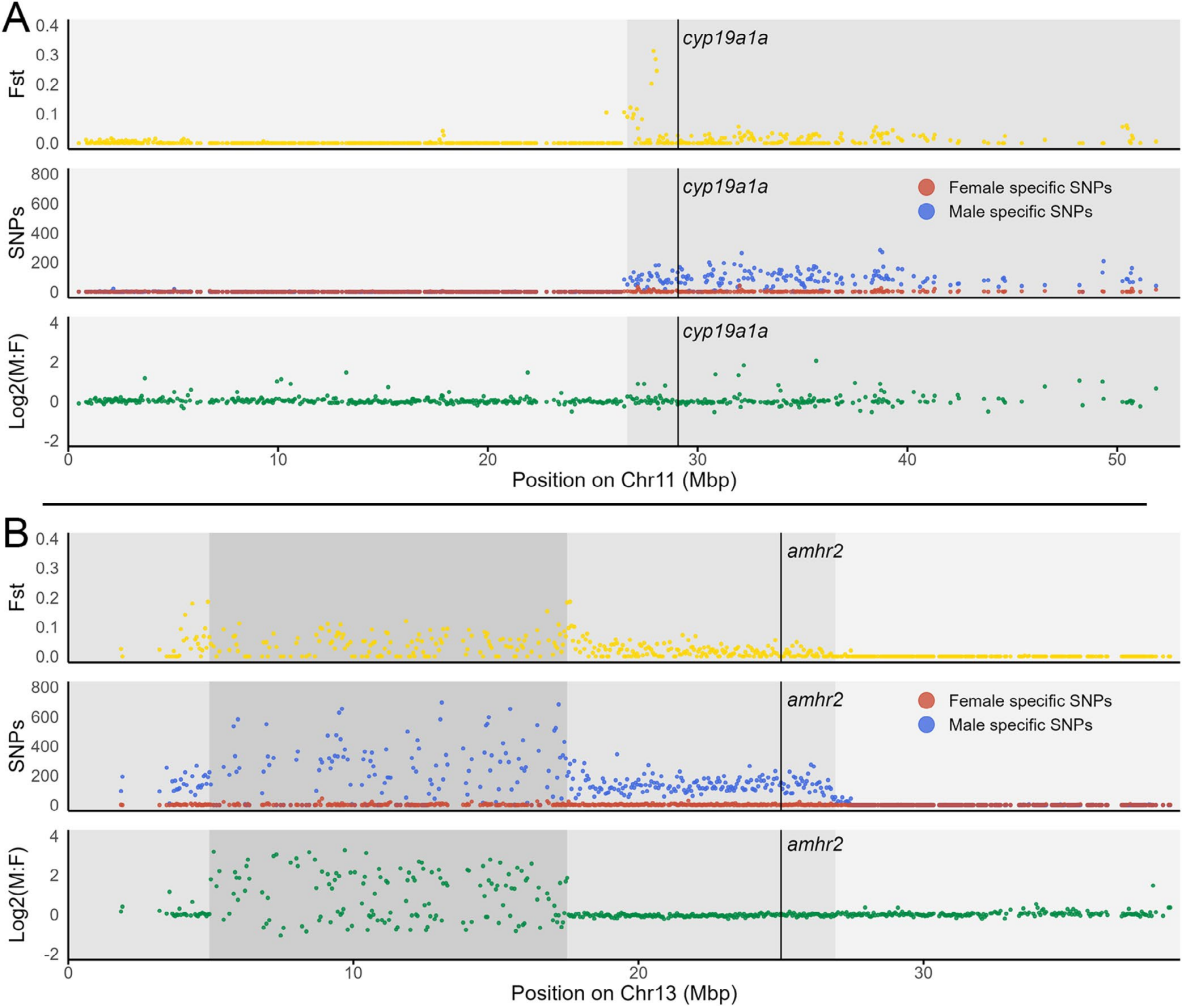
Sex‐linked regions in *N. guentheri* . Plots show results of Pool‐seq analyses [male and female specific SNPs and relative divergence (F_ST_) are in blue, red and yellow, respectively] and coverage (in green) using pseudochromosomes constructed with *N. furzeri* as a reference. (A) chromosome 11; (B) chromosome 13. Grey shading marks putative boundaries of the evolutionary strata along each chromosome. Note that these chromosomes correspond to X_1_ and X_2_ chromosomes, which pair with the neo‐Y chromosome of fusion origin. Results for all chromosomes are shown in Figure S3.

To identify sex‐linked regions, male and female Pool‐seq data of *F. thierryi* and *N. guentheri* were mapped to the corresponding reference RaGOO pseudochromosomes assembled using the *N. furzeri* genome. Pool‐seq analyses identified putative pseudoautosomal regions with no differentiation as well as regions of increased F_ST_ between males and females, and male‐specific SNPs on chromosomes 3 and 14 in *F. thierryi* (Figure 1 and Figure S2) and chromosome 11 and 13 in *N. guentheri* (Figure 2 and Figure S3). Regions of increased F_ST_ extended beyond regions of increased male‐specific SNPs on both *F. thierryi* Chr3 and Chr14 (Figure 1), which suggests the presence of non‐recombining strata with distinct levels of differentiation. A similar pattern was also observed on *N. guentheri* Chr13 (Figure 2). We would like to highlight that given the genomes were scaffolded using the *N. furzeri* genome as a reference, the synteny blocks which are linked by a fusion forming the large Y chromosome in *F. thierryi* and *N. guentheri* are not fused in *N. furzeri* , and that is why they look like unlinked regions.

Pool‐seq data analyses were complemented by coverage analyses comparing sequencing depth between sexes (Figures 1 and 2; Figures S2 and S3), which can identify well‐differentiated sex‐linked sequences. In *F. thierryi* , no differences in coverage were observed between sexes, which suggests its multiple sex chromosome system is quite young. However, the analysis identified a well‐differentiated region on Chr13 in *N. guentheri* (Figure 2), clearly supporting the presence of two strata of different ages in the non‐recombining region.

### 3.3 Analyses of the Putative Sex‐Determining Regions in *F. thierryi* and *N. guentheri*

**FIGURE 3:**
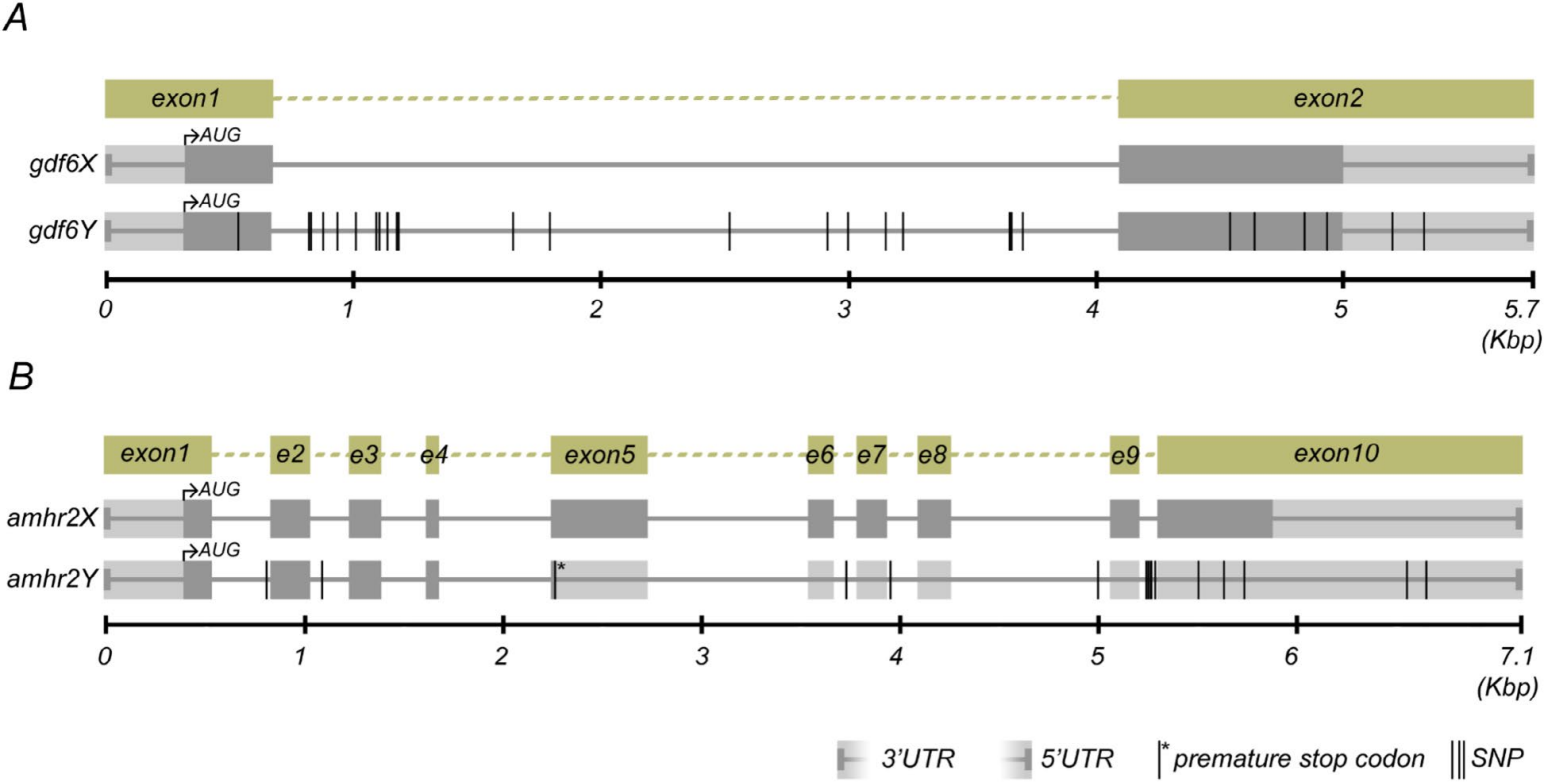
Schematic representation of putative sex‐determining genes *gdf6* and *amhr2* in *F. thierryi* and *N. guentheri* , respectively. (A) *F. thierryi gdf6X* and *gdf6Y* alleles with intron‐exon boundaries following the *N. furzeri* gene model XM_015949385.2. (B) *amhr2X* and *amhr2Y* gametologs with intron‐exon boundaries delimited using the *N. furzeri* *amhr2* consensus model (merged cds for KAF7209949.1 and GAIB01133837 transcript) as a reference. For details on SNPs see Dryad repository (Hospodářská et al. 2024).

From all common SD genes analysed in the primary screening, *hsd17b* and *gdf6* were found to be sex‐linked in *F. thierryi* and *amhr2* in *N. guentheri* (Figures 1 and 2; Table S1). The *hsd17b* gene was localised in an undifferentiated, possibly pseudoautosomal region of the *F. thierryi* pseudochromosome 3 (chr3: 7,547,266–7,550,587). Accordingly, none of the two SNPs and one 2 bp indel identified in the male sequence was male specific as all variants were found also in the female dataset, albeit the indel only upon manual inspection (Dryad repository; Hospodářská et al. 2024). The *F. thierryi gdf6* gene, however, was localised in a differentiated region of pseudochromosome 3 with increased F_ST_ and male specific SNPs (chr3: 33,044,167–33,049,935). Upon manual curation, we identified 27 Y‐specific SNPs, five of them in exons (Figure 3 and Dryad repository; Hospodářská et al. 2024). However, only one substitution in exon 1 (chr3: 33,044,706 A>G) was non‐synonymous, and comparison with the *N. furzeri* GDF6X and GDF6Y protein sequences showed that the amino acid encoded by the Y‐linked variant is shared and therefore likely ancestral (Dryad repository; Hospodářská et al. 2024).

In *N. guentheri* , *amhr2* was localised on pseudochromosome 13 (chr13: 24,999,373‐25,006,519). We found discrepancies in intron‐exon structure between the *amhr2* gene annotated in the *N. furzeri* genome (cf. coding sequence for the protein KAF7209949.1) and transcript GAIB01133837 from the *N. furzeri* transcript catalogue by Petzold et al. (2013), suggesting that the gene model is not complete. We merged both records into a consensus model used as a reference in downstream analyses of the *N. guentheri amhr2* (Figure 3, Dryad repository; Hospodářská et al. 2024).

Further analysis of the Pool‐seq reads mapped to the *amhr2* sequence uncovered four variable sites in the female dataset and 18 variable sites in the male dataset (Dryad repository; Hospodářská et al. 2024). However, manual inspection of the female variants showed these correspond to errors in the reference sequence (see below), which either contains Y‐linked SNPs or a missing repeat motif in di‐ and pentanucleotide repeats. Thus, no reliable SNP was found in the female dataset (Figure 3). Similarly, we excluded two male variants upon manual inspection (Figure 3). Out of 16 male‐specific SNPs found in the *N. guentheri* *amhr2* gene, only four were localised in exons (Figure 3 and Dryad repository; Hospodářská et al. 2024). The most prominent variant, namely T>C substitution in position 25,001,642 on the pseudochromosome 13, coincides with exon 5 of the *amhr2* according to the consensus gene model (Figure 3 and Dryad repository; Hospodářská et al. 2024). Comparison of the compositions of the alignment stack at the target position showed that in females all 46 reads carry the “C” variant, while in 59 male reads we observed a 34% “T” to 66% “C” split (Dryad repository; Hospodářská et al. 2024). This suggests that the “C” variant represents the X‐linked allele, while the “T” variant is Y‐linked. Overlaying this position with the consensus gene annotation, we can see that the T>C substitution causes a premature stop codon in exon 5, which is missing in the X‐linked allele (Figure 3).

Trio binning assembly using a selected set of reads mapping to *N. guentheri* pseudochromosome 13 yielded two haplotypes (Dryad repository; Hospodářská et al. 2024). The trio‐binned maternal haplotype supported the female‐specific variant inferred from the Pool‐seq data. The paternal haplotype contained two contigs bearing partial *amhr2* sequences and supported the specificity of the Y‐linked T>C substitution encoding the stop codon. The substitution was confirmed also by the duplex PCR assay (Figure S4) in 15 NGU1 males and all four tested males from the population NGU2. However, five NGU1 males lacked the Y‐specific amplicon (designated as *amhry‐*). All 11 females had only a single PCR band as expected. Sanger sequencing confirmed the X‐allele variant in the polymorphic position in all five *amhry‐* males. To investigate further the possible reasons behind this unusual pattern, we analysed the chromosomal material available for two *amhry‐* males (see below).

### 3.4 BAC‐FISH

We screened the *N. furzeri* BAC library for clones bearing common fish sex‐determining genes and confirmed their presence by PCR (Table S2) and Oxford Nanopore sequencing (see the Dryad repository). The verified clones (Table 2) were hybridised to species under study (Figures S5 and S6). In outgroup species, *F. thierryi* , we identified *gdf6* as the possible MSD candidate, similarly as was reported in *N. furzeri* and *N*. *kadleci* (Reichwald et al. 2015; Štundlová et al. 2022; Richter et al. 2025). This also corresponds to results observed from Pool‐seq analysis, which revealed that multiple sex chromosomes of *F. thierryi* correspond to Chr14 and Chr3 (sex chromosome) of *N. furzeri* (Figure 1). Thus, they share the MSD gene *gdf6*, located on Chr3. On the contrary, in all other tested species, the BAC clone with the *gdf6* gene hybridised to autosomes. In both species from the Coastal clade, *N. guentheri* and *N. lourensi* , only the gene *amhr2* hybridised to sex chromosomes and thus seems to be a promising MSD candidate in this particular clade. Also here, our results from Pool‐seq analysis in *N. guentheri* correspond with the localisation of *gdf6* on autosomes and not on sex chromosomes. Similarly, in *N. brieni* (Kalahari clade) we localised *amhr2* on sex chromosomes. However, none of the tested SD genes (Table 2) hybridised to sex chromosomes of *N*. *ditte* (also Kalahari clade) (Figure S6). The data hence point to the presence of at least two candidate MSD genes in the Kalahari clade: *amhr2* and one another, so far unknown. Finally, it is important to note that in *N. brieni* and *N. lourensi* (Figure S5L',M',U',V'), we observed four signals after BAC‐FISH with the clone containing the *gdf6* gene. Reprobing with the FTH‐triv painting probe allowed us to infer that a weaker pair of signals reside on the synteny blocks harbouring the original *gdf6* locus, while the remaining, stronger pair of signals might represent false positives due to repetitive sequences contained on the selected BAC clone.

### 3.5 Basic Chromosomal Patterns and WCP Probes Validation

We confirmed previously reported diploid chromosome numbers and the presence of sex chromosomes in all species under study (Krysanov and Demidova 2018). Except for *N. lourensi* and *N. guentheri* population NGU2, both sexes from the same populations have been cytogenetically analysed in our previous studies (Štundlová et al. 2022; Lukšíková et al. 2023; Voleníková et al. 2023).

The three sex chromosome‐specific painting probes prepared for *F. thierryi* , *N. guentheri* , and *N. lourensi* were first hybridised back onto the male metaphases of the respective species to confirm the probe specificity. Each probe painted, from end to end, all three elements of the X_1_X_2_Y system in each given species (Figure S5). It is worth mentioning that the NGU‐Y probe confirmed the identity and composition of multiple sex chromosomes in both studied *N. guentheri* populations which, however, differ by their 18S rDNA FISH patterns (Figure S1C,D).

### 3.6 Detection of Chromosomal Homeologies by Zoo‐FISH Experiments

**FIGURE 4:**
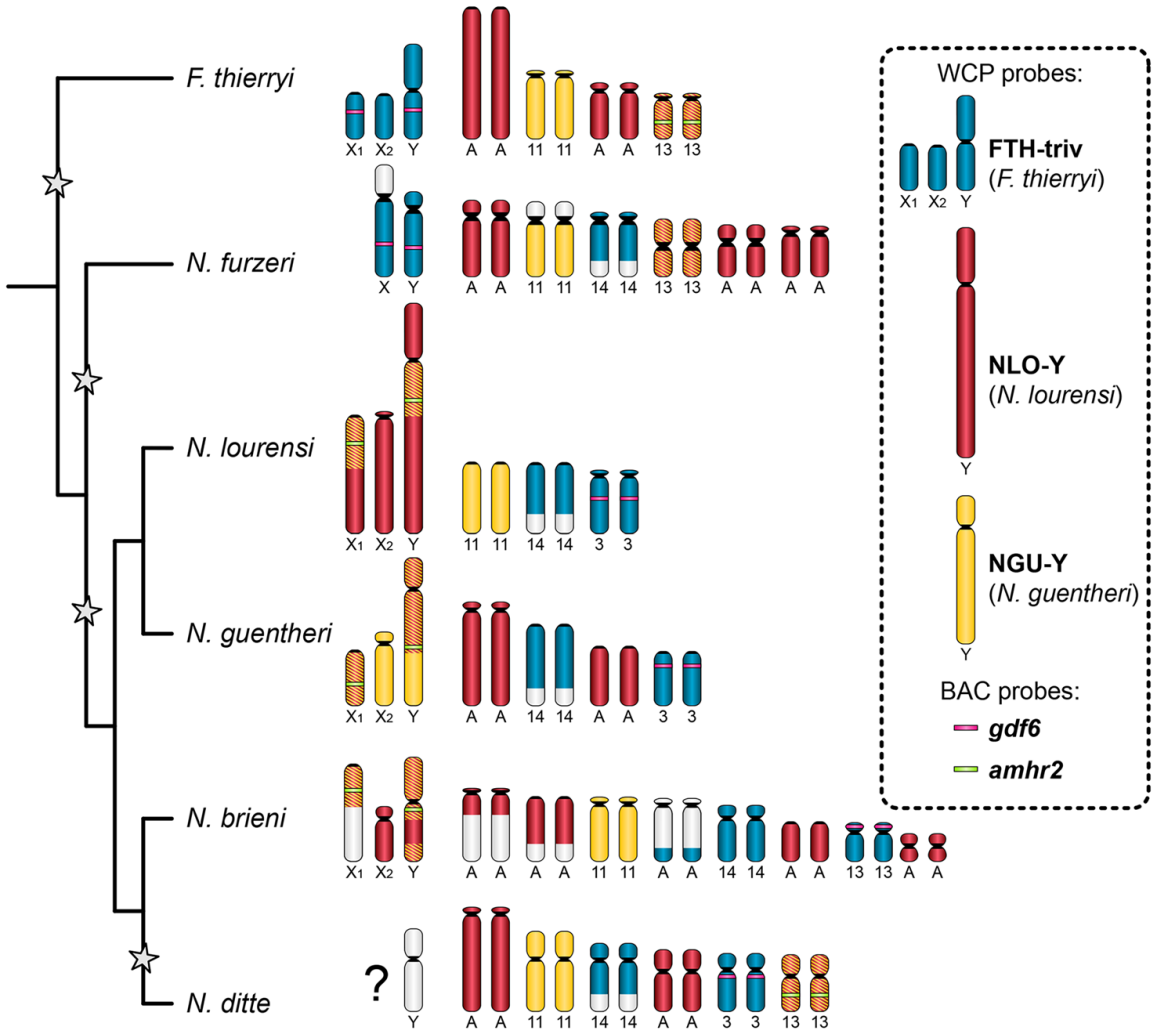
Schematic overview of WCP and BAC‐FISH hybridization patterns. The content and colour coding of WCP and BAC‐FISH probes is depicted on the right panel. FTH‐triv = meiotic sex trivalent from *F. thierryi* ; NLO‐Y = Y chromosome from *N. lourensi* ; NGU‐Y = Y chromosome from *N. guentheri* . Segments in crosshatched red‐yellow colour represent overlap between the NGU‐Y and NLO‐Y probes. Note that none of the WCP probes hybridised to X_1_X_2_Y sex chromosomes of *N. ditte*. *Gdf6* and *amhr2* = candidates for sex‐determining gene contained in the mapped BAC clones; their approximate location is depicted on sex chromosomes and, where unambiguously assigned to certain chromosome pairs, also on autosomes. Relative position of chromosome markers has been assessed based on metric analysis performed on three metaphases per species (Tables S6–S11), independently for each marker. Where possible, we numbered the autosome pairs according to *N. furzeri* genome reference (Willemsen et al. 2020), with chromosome pairs labelled with FTH‐triv probe corresponding to NfuChr3 and NfuChr14 (a pair with and without *gdf6*, respectively), and NGU‐Y corresponding to NfuChr13 and NfuChr11 (a pair with and without *amhr2*, respectively). Remaining autosomes are marked as “A”. Autosomes carrying false‐positive signals with *gdf6*‐bearing BAC clone (see BAC‐FISH results above and Figure S5) are not included. In *N. brieni* , a large accumulation of constitutive heterochromatin has taken place on the long arm of X_1_ chromosome; synteny of this segment to the remainder of the *N. brieni* genome could not be assessed. Phylogenetic relationships follow van der Merwe et al. (2021) and, for *N. lourensi* , Bartáková et al. (2025). Asterisks mark the proposed four independent origins of *Nothobranchius* sex chromosomes.

All hybridization patterns are provided in Figure S5 and schematized in Figure 4. The WCP probes revealed unambiguously the identity of mapped synteny blocks across the set of studied species. Only the NLO‐Y probe produced additional signals on *N. brieni* male chromosomes (see below) and the NGU‐Y probe produced a few additional signals on varied numbers of autosomes in different species due to the presence of major rDNA clusters on the Y and X_1_ chromosome, as was revealed by rDNA FISH experiments (see Figure S1C,D).

In *F. thierryi* , each painting probe stained different chromosomes but the NGU‐Y and NLO‐Y probes entirely overlapped with their signal patterns on a single pair of small acrocentric chromosomes. While the NGU‐Y probe painted four chromosomes, the NLO‐Y probe stained six of them.

In *N. furzeri* , the FTH‐triv and NGU‐Y probes both produced signals on four chromosomes, with non‐overlapping distribution patterns. The probes, however, left large (peri)centromeric regions unstained, due to different repetitive DNA content, as may be inferred from our previous study (Voleníková et al. 2023). The FTH‐triv probe painted a pair of small acrocentric chromosomes and the long arms of XY sex chromosomes, which were clearly heteromorphic, in line with our previous report (Štundlová et al. 2022). The NLO‐Y probe generated eight distinct signals together with a number of additional putative signals (including the one on the Y chromosome, denoted by asterisk) which may reflect shared repetitive DNA content (Štundlová et al. 2022; Lukšíková et al. 2023).

In *N. lourensi* , the FTH‐triv probe entirely painted four acrocentric autosomes. The NGU‐Y probe marked, besides a pair of other acrocentric autosomes, also distinct segments of two sex chromosomes, where the signal overlapped with the NLO‐Y probe. Specifically, the two probes co‐localised in the segment placed proximally on the long arms, covering about one fourth of the Y‐chromosome length. Complementary experiments by BAC‐FISH showed that this segment contains the *amhr2* gene. The homologous segment was placed proximally on a medium‐sized acrocentric sex chromosome, covering the proximal half of this chromosome. Given the presence of this segment, the sex chromosome was denoted as X_1_.

The signal patterns in *N. guentheri* were as follows: the FTH‐triv probe hybridised entirely to four acrocentric autosomes. The NLO‐Y probe co‐localised with the NGU‐Y probe in the proximal half of the Y chromosome and over the entire length of the smaller one from the two acrocentric X chromosomes. Given its content (together with the confirmed presence of *amhr2* locus by BAC‐FISH), this chromosome was denoted as X_1_. The location of *amhr2* on the Y chromosome was very close to the fusion breakpoint, which could be inferred based on the rDNA signal pattern (see Figure S1C,D). This region could be sometimes apparent as an unlabelled constriction on the long Y chromosome arms (Figure S5).

We further analysed the two *amhry‐* males by means of WCP with the NGU‐Y probe and dual‐colour FISH with 5S and 18S rDNA as markers residing on sex chromosomes of this species. In both male individuals, we observed a female constitution with 2n = 36 and two pairs of sex chromosomes, most likely corresponding to X_1_X_1_X_2_X_2_ sex chromosome constitution. Chromosomes in meiotic prophase I (diplotene/diakinesis) were paired in 18 standard bivalents and no sex trivalent was observed (Figure S7; compare with Figure S1C).

Regarding *N. brieni* , the most informative was the NGU‐Y probe which, besides staining again a pair of acrocentric autosomes, painted the majority of the Y chromosome, leaving a narrow centromere‐proximal region on the long arms unstained. A probable X_1_ sex chromosome had only the proximal one third of its length marked by the NGU‐Y probe, while the rest of the long arms was composed of a DAPI‐positive block. The NLO‐Y probe painted the entire Y chromosome, including the narrow NGU‐Y‐negative gap. NLO‐Y and NGU‐Y probes also co‐localised on the proximal half of X_1_. Additionally, the NLO‐Y probe produced the signal patterns over many other chromosomes, some of which probably reflect rather repetitive content than identity of synteny blocks. Based on the Y‐linked patterns and sequential C‐banding (Lukšíková et al. 2023 and this study; Figure S1E,F), one of the painted small chromosomes is X_2_. Integrated WCP/BAC‐FISH experiments confirmed the placement of *amhr2* in the (peri)centromeric region of the Y, and proximally on the long arms of X_1_. The FTH‐triv probe labelled one pair of small submeta‐to‐subtelocentric chromosomes, long arms of a pair of medium‐sized subtelocentrics, and a narrow terminal portion of long arms on the pair of another medium‐sized subtelocentrics.

Zoo‐FISH in *N. ditte* produced exceptional patterns in the way that none of the applied probes matched the metacentric Y chromosome. While X chromosomes could not be unambiguously identified, it may be inferred that the hybridisation signals are absent also from them. The FTH‐triv probe entirely painted one medium‐sized submetacentric pair, and most of the length (except for a narrow terminal part on the long arms) of the medium‐sized subtelocentric pair. The NGU‐Y probe stained two submetacentric pairs, with the NLO‐Y probe co‐hybridising to the smaller of these pairs, but only across its long arms. Besides that, the NLO‐Y probe entirely painted one large and one small acrocentric pair.

Metric analyses performed on three metaphases per species enabled a comparison of relative values for chromosomal homeologies and the localisation of the *gdf6* and *amhr2* genes. The resulting data were used to create a schematic overview (Figure 4). Detailed measurements and values for the informative chromosomes carrying the markers under study are provided in Tables S6–S11.

## 4 Discussion

In this work, we studied four *Nothobranchius* killifish species and their outgroup *F. thierryi* , all having the X_1_X_2_Y sex chromosome system, using a combination of cytogenetic and genomic approaches. Given the high incidence of multiple sex chromosomes of presumably independent origin in the genus (*N* = 6), equalled among teleosts only by Neotropical *Harttia* catfishes (Deon et al. 2020; Sember et al. 2021), and the well‐resolved phylogenetic relationships (van der Merwe et al. 2021; Bartáková et al. 2025), *Nothobranchius* killifishes are a highly informative lineage for investigating evolutionary forces that drive sex chromosome turnovers (Palmer et al. 2019; Vicoso 2019; Sember et al. 2021; Kitano et al. 2024). The present study provides, for the first time, empirical evidence of sex chromosome turnover in *Nothobranchius* killifishes, confirming thus previous speculations based on indirect evidence (Krysanov and Demidova 2018; Simanovsky et al. 2019; Lukšíková et al. 2023).

We revealed at least four independent origins of sex chromosomes in this genus and outgroup *F. thierryi* (Figure 4). FISH with WCP probes (Figure 4 and Figure S5) confirmed that the *Nothobranchius* multiple sex chromosome systems under study evolved by Y‐autosome fusion (cf. Krysanov et al. 2016; Krysanov and Demidova 2018; Simanovsky et al. 2019). The large subtelocentric Y chromosome of *N. lourensi* originated from subsequent fusions of three different synteny blocks. Despite the independent autosomal additions, X_1_X_2_Y systems of *N. guentheri* , *N. brieni* , and *N. lourensi* shared a synteny block, which suggests the presence of an ancestral sex chromosome pair, originating from the common ancestor of these three species. This system would therefore precede the split between Coastal and inland clades, the latter encompassing Kalahari clade, dated around 9–11 MYA (van der Merwe et al. 2021). It seems reasonable to assume that other closely related *Nothobranchius* species within these clades possess the same sex chromosomes which just cannot be detected cytogenetically. To test the homeology of the putative ancestral sex chromosome synteny block, it will be critical to compare patterns of sequence divergence and calculate divergence times between sex chromosomes of these related species (cf. Charlesworth 2021; Li et al. 2021; Sardell et al. 2021).

The shared sex‐linked synteny block could alternatively suggest independent co‐option of the same chromosome for a role in sex determination. We showed that previously characterised XY sex chromosomes of *N. furzeri* and *N. kadleci* (Reichwald et al. 2015; Willemsen et al. 2020; Štundlová et al. 2022; Richter et al. 2025) share a partial homology with the X_1_X_2_Y system of *F. thierryi* . The shared segment carries the *gdf6* gene which makes it a promising candidate MSD in *F. thierryi* . If confirmed, *gdf6* was likely co‐opted independently from the system which evolved in *N. furzeri* and *N. kadleci*. The shared origin is unlikely as the sequence divergence analysis between *gdf6X* and *gdf6Y* alleles in *N. furzeri* and *N. kadleci* places the emergence of their XY system shortly before the split of these species, after their divergence from *N. orthonotus* (Štundlová et al. 2022; Richter et al. 2025). The possible independent co‐option would point to phylogenetic constraint in sex determination of killifishes as *gdf6* has been thus far identified as putative MSD only in the Pachón cave population of blind tetra *Astyanax mexicanus* (Imarazene et al. 2021) and it is among potential candidates for MSD in Greenland halibut (Ferchaud et al. 2022). Nevertheless, it should be noted that the same synteny block co‐opted as a sex chromosome in different species may harbour different loci with the sex‐determining role, as showed for example, in *Takifugu niphobles* (Ieda et al. 2018).

We also demonstrated that X_1_X_2_Y sex chromosomes of *N. ditte* evolved independently from yet another linkage groups, which are not involved in sex chromosomes of any other studied *Nothobranchius* species. The sex in this species is therefore likely determined by yet another MSD. We tested most of the usual suspects for the MSD role in teleosts by means of BAC‐FISH (Herpin and Schartl 2015; Pan et al. 2021). Sex in *N. ditte* thus could be determined either by *bmpr1* for which we had no BAC clone, a less common mechanism, or a gene duplicate (cf. Kitano et al. 2024; Wang et al. 2024).

Chromosome inversions are instrumental for restriction of recombination and were invoked to explain the polymorphic Y chromosome in *N. furzeri* and *N. kadleci* populations (Reichwald et al. 2015; Štundlová et al. 2022). The Y chromosome of *N. brieni* also evolved through fusion and subsequent inversion, which was indicated by discontinuity of segments painted by the NGU‐Y probe. Furthermore, the comparison of the hybridisation patterns of the *amhr2* BAC and rDNA clusters on X_1_ and the Y chromosome of *N. guentheri* (Figures S1 and S5) suggested subsequent small‐scale inversion(s) following the original fusion, which shifted the order of these loci and the position of the centromere on the Y chromosome.

Independent sex‐linked inversions can give rise to evolutionary strata, that is, regions which stopped recombining at different evolutionary time points (e.g., Lahn and Page 1999; Zhou et al. 2014). Evolutionary strata have been already reported in teleosts, for example, in guppies (reviewed in Darolti et al. 2022) and sticklebacks (Peichel et al. 2020; Sardell et al. 2021). To determine the degree of sex chromosome differentiation and the extent of non‐recombining regions, we performed long‐read sequencing of the *F. thierryi* female and *N. guentheri* male genomes, combined with male vs. female coverage and Pool‐seq data analyses (Figures 1 and 2; Figures S2 and S3). As for the difference in coverage between male and female genomes, the sequences have not yet diverged enough in *F. thierryi* , pointing to a low‐to‐moderate level of differentiation in the non‐recombining region. In *N. guentheri* , however, our analyses showed two clearly defined regions on Chr13 enriched in male‐specific SNPs, which differed in their degree of sequence divergence between gametologs (Figure 2). This is suggestive of the presence of two evolutionary strata. The older stratum may correspond to the ancestral sex‐linked chromosome shared by the Coastal and inland clades (see above).

MSD genes are frequently recruited from members of the TGF‐β signalling pathway in teleost fishes (Pan et al. 2021; Kitano et al. 2024; Wang et al. 2024). In the *F. thierryi* genome, we annotated two sex‐linked genes serving as MSD in other fishes (Pan et al. 2021), namely *gdf6* and *hsd17b1*. The sole presence of “usual suspect” on the sex chromosome, however, does not a priori imply its role as MSD (e.g., *gsdf* in *Sebastes* rockfishes; Sykes et al. 2023). The *hsd17b1* gene is localised in the putative pseudoautosomal region of Chr3 with no differentiation (Figure 1 and Table S1) and shows no allelic variation (Dryad repository; Hospodářská et al. 2024). However, the *F. thierryi* *gdf6* gene is localised within the putative non‐recombining region of Chr3, as evidenced by increased F_ST_ and male‐specific SNPs (Figure 1; Table S1). Yet, there is only one non‐synonymous change located in exon 1. The Y‐linked variant encodes an amino acid that is shared with the protein products of both *N. furzeri* *gdf6X* and *gdf6Y* alleles. Additionally, in *N. furzeri* , non‐synonymous substitutions affecting protein interactions are localised at the protein's C‐terminus, that is, the opposite end of the gene (Reichwald et al. 2015). Hence, *gdf6* seems unlikely to be the primary sex‐determining gene in *F. thierryi*. If it is responsible for sex determination in *F. thierryi* , it would probably act via mutations in the regulatory sequences that alter its expression profile, rather than through changes in its coding regions. An altered spatio‐temporal expression profile has been described for some teleost MSD genes, for example, in medaka *Oryzias luzonensis* (Takehana et al. 2014), sablefish *Anoplopoma fimbria* (Herpin et al. 2021), and Atlantic halibut *Hippoglossus hippoglossus* (Edvardsen et al. 2022).

The *amhr2* gene is sex‐linked in *N. guentheri* as it is located on Chr13 (Figure 2 and Table S1), thus representing a candidate MSD gene (cf. Feron et al. 2020; Nakamoto et al. 2021; Qu et al. 2021; Wen et al. 2022). Our results suggest that a mutation in exon 5 of the Y‐linked *amhr2* allele produces a stop codon and thus encodes for a truncated protein (Figure 3). Experimental knock out of *amhr2* in teleosts led to male‐to‐female sex reversal as exemplified by medaka *hotei* mutant (Morinaga et al. 2007; Nakamura et al. 2012), Nile tilapia (Li et al. 2015), and ayu (Nakamoto et al. 2021). It should be stressed that in these cases both *amhr2* alleles were inactivated. The *N. guentheri* sex determination mechanism may be similar to those reported in the yellow perch *Perca flavescens* and the catfish family Pangasiidae in which the Y‐linked *amhr2* duplicate is truncated in its N‐terminus (Feron et al. 2020; Wen et al. 2022; Kuhl et al. 2024). We, however, did not detect any other paralog of the *amhr2* gene in the genome of *N. guentheri* , although we cannot exclude recent duplicates to be collapsed in our assembly, which clearly lacks X‐ and Y‐linked haplotypes of the non‐recombining region. Nevertheless, delimitation of exon 5 needs to be confirmed. Moreover, *amhr2* is localised in the younger of the two *N. guentheri* Chr13 strata (Figure 2) and shows very little allelic differentiation, which is lower than the divergence of sex‐linked *gdf6* alleles in *N. furzeri* (Štundlová et al. 2022). This suggests that differentiation of the *N. guentheri* *amhr2* alleles started less than 2 MYA (cf. Štundlová et al. 2022). Therefore, it seems unlikely that the *amhr2* gene represents the sex determination system associated with the presumed ancestral sex chromosome pair shared by *N. guentheri* , *N. brieni* and *N. lourensi* (see above). Genes annotated in the older stratum do not provide any other clear candidate for MSD in *N. guentheri* , suggesting its possible pseudogenisation upon sex chromosome turnover.

Interestingly, the *N. guentheri* *amhr2y‐* males with typical gonad morphology and male body coloration, yet female karyotype, suggested possible female‐to‐male sex reversal. This situation where environmental cues occasionally override genetic sex determination has been described in several teleosts (e.g., Shen and Wang 2019) but so far has never been observed in *Nothobranchius* killifishes.

We searched the *F. thierryi* and *N. guentheri* genomes also for other genes from the teleost sex determining pathway (Kitano et al. 2024). The *rspo1*, *cyp11b*, and *wnt4* genes are sex‐linked in *F. thierryi* , while the *cyp19a1a* gene encoding an aromatase is sex‐linked in *N. guentheri* (Figures 1 and 2; Table S1). It was hypothesised that sex chromosome turnover via sex chromosome‐autosome fusions can be driven by selection for linkage between a sex chromosome and an autosome, which resolves sexual conflict (Charlesworth and Charlesworth 1980; Kitano et al. 2009, 2024; Pennell et al. 2015). It is intriguing to speculate that fixation of the *F. thierryi* and *N. guentheri* multiple sex chromosome systems was facilitated by linkage between MSD and genes with sex‐related function (cf. Pala et al. 2012; Sigeman et al. 2019) and it surely warrants further study of genomic distribution of sex‐related genes (Sigeman et al. 2019) and genes with sex‐biased expression in killifishes (cf. Toups et al. 2019; Lichilín et al. 2021; Mora et al. 2024).

Male‐determining regions in *N. brieni* and *N. guentheri* are located close to the centromere and/or assumed rearrangement breakpoints on their Y chromosomes (Figure 4). An increasing number of studies demonstrated significantly reduced recombination around the chromosome fusion breakpoints in various taxa (Gil‐Fernández et al. 2020; Vara et al. 2021; Yoshida et al. 2023; MacLeod‐Bigley and Boulding 2023; Lisachov et al. 2023; Xu et al. 2024), and several lines of cytogenetic or genomic evidence suggest this might have contributed to sequence differentiation of multiple sex chromosomes of some fish species (de Moraes et al. 2019; Li et al. 2021; Sardell et al. 2021; Huang et al. 2025). The effect of suppressed recombination might be further enhanced by the close proximity to the centromere as pericentromeric regions display low recombination rates (Nambiar and Smith 2016; Miller and Hawley 2017; Filatov 2024). Recombination rates are also low in the central regions of large chromosomes (Haenel et al. 2018). All these mechanisms might have contributed to the differentiation of *Nothobranchius* sex‐determining Y‐linked regions. In *N. guentheri* , Y‐autosome fusion might have formed the younger stratum bearing the *amhr2* gene adjacent to the breakpoint, while the older stratum representing putative ancestral SD region could predate the fusion. The *N. brieni amhr2* gene is also localised between a centromere and a fusion breakpoint. The presence of sex‐determining regions in the interstitial position on the large Y chromosomes may substantially reduce or even abolish recombination in species with heterochiasmy, that is, a sex‐specific pattern of recombination with crossovers limited to chromosome ends in males but being equally distributed in females (Sardell and Kirkpatrick 2020). Heterochiasmy was reported in *N. furzeri* and *N. kadleci* and suggested to be responsible for reduced recombination between X and Y sex chromosomes (Štundlová et al. 2022). Hence, the proximity to the centromere and rearrangement breakpoints as well as heterochiasmy should be considered the important drivers in the establishment of the SD region. Further studies are needed to assess whether the evolution of different X_1_X_2_Y systems in *Nothobranchius* was affected by recombination cold spots.

## 5 Conclusions

*Nothobranchius* sex chromosomes are highly dynamic and evolved repeatedly from different linkage groups. Taking into account the availability of well‐resolved phylogeny, this fish genus represents an excellent model system to investigate fundamental questions related to sex chromosome evolution and turnover. Our findings collectively suggest at least four independent origins of sex chromosomes in the killifishes under study. We also delineated regions of suppressed recombination and identified candidates for MSD genes in *F. thierryi* and *N. guentheri* . We suggest that recombination cold spots such as centromeric regions and fusion breakpoints can facilitate recombination suppression and subsequent differentiation of sex determining regions. Our study highlights the need for combined cytogenetic and genomic methodologies in analysing sex chromosomes, as they are mutually informative and allow us to draw a more complete picture of sex chromosome evolution.

## Author Contributions

Conceptualisation: A.S., P.N.; data curation: P.N.; formal analysis: M. Hospodářská, P.M., A.C.V., P.N.; funding acquisition: A.S.; investigation: M. Hospodářská, P.M., A.C.V., A.A.‐R., S.A.S., T.P., M.A., K.J., J.Š., N.T., K.B., M.J., M. Hiřman, T.L., E.Y.K., P.N., A.S.; methodology: A.S., P.N., A.C.V., A.A.‐R., T.L.; project administration: A.S., P.N.; resources: A.S., P.N., M.R., C.E., P.R., T.L., S.A.S.; supervision: A.S., P.N., M.R.; validation: M. Hospodářská, P.M., A.C.V., M.A., A.S., N.T., P.N.; writing original draft: A.S., M. Hospodářská, P.N., P.M., N.T.; writing – review and editing: P.N., A.S., P.M., M.A., N.T., M.R., A.C.V., M. Hospodářská, M.J., J.Š., C.E., P.R., S.A.S., E.Y.K., T.L.

## Disclosure

*Benefit Sharing Statement*: The worldwide scientific community will benefit from the open sharing of the data presented in this manuscript.

## Ethics Statement

The experimental part involving fish individuals was supervised by the Institutional Animal Care and Use Committee of the Institute of Animal Physiology and Genetics CAS, v.v.i., with the supervisor's permit number CZ 02361 certified and issued by the Ministry of Agriculture of the Czech Republic. The handling of fish individuals to obtain chromosomes followed European standards in agreement with §17 of the Act No. 246/1992 coll. The experiments with *N. brieni* , *N. ditte*, and one population of *N. guentheri* (NGU2) were approved by the Ethics Committee of Severtsov Institute of Ecology and Evolution (Order No. 27 of November 9, 2018). In case of chromosome preparations from the kidneys and gonads, fishes were euthanised before organ sampling using 2‐phenoxyethanol (Sigma‐Aldrich, St. Louis, MO, USA). A narrow stripe of the tail fin was taken from live specimens after fishes were anaesthetised by MS‐222 (Merck KGaA, Darmstadt, Germany).

## Conflicts of Interest

The authors declare no conflicts of interest.

## Supporting information


Data S1.

## Data Availability

Raw sequencing data generated in this project are available at the Sequence Read Archive hosted by the National Center for Biotechnology, under the BioProject accession no. PRJNA1286812. Genome assemblies and annotation files as well as other processed datasets were deposited in the Dryad digital data repository (Hospodářská et al. 2024). All other relevant data are presented in the article and its (Supporting Information).
