# Supplementary material for: Multiple Origins of Sex Chromosomes in Nothobranchius Killifishes
Source: Mol Ecol. 2025 Jul 24;34(16):e70029. doi: 10.1111/mec.70029 (PMC12329641; doi:10.1111/mec.70029)
Supplement: Supplementary file 1 — Data S1. [file MEC-34-e70029-s001.pdf]

Supporting Information for

Multiple Origins of Sex chromosomes in *Nothobranchius* Killifishes

Journal: Molecular Ecology

Monika Hospodářská, Pablo Mora, Anna Chung Voleníková, Ahmed Al-Rikabi, Marie Altmanová, Sergey A. Simanovsky, Nikolas Tolar, Tomáš Pavlica, Karolína Janečková, Jana Štundlová, Kseniya Bobryshava, Marek Jankásek, Matyáš Hiřman, Thomas Liehr, Martin Reichard, Eugene Yu. Krysanov, Petr Ráb, Christoph Englert, Petr Nguyen*, Alexandr Sember*

* Correspondence:

sember@iapg.cas.cz; Institute of Animal Physiology and Genetics, Czech Academy of Sciences, Liběchov, Czech Republic

petr.nguyen@entu.cas.cz; Institute of Entomology, Biology Centre of the Czech Academy of Sciences, České Budějovice, 370 05, Czech Republic

The PDF file includes:

Table S1
Table S2
Table S3
Table S4
Table S5
Table S6
Table S7
Table S8
Table S9
Table S10
Table S11
Figure S1
Figure S2
Figure S3
Figure S4
Figure S5
Figure S6
Figure S7

Table S1. Genes of teleost sex determining pathway used to screen for BAC clones and searches in the *N. guentheri* (Ngu) and *F. thierryi* (Fthi) genomes including their genome locations.

Gene	ID	Ngu primary assembly	Ngu RaGOO pseudochromosomes	Fthi primary assembly	Fthi RaGOO pseudochromosomes
amh	EF512167.1	scaffold_219:26008468-26008070	chromosome17_RaGOO:6760386-6760784	contig_4730:17760-17536	chromosome17_RaGOO:7014102-7013878
amhr2	AB618627.1	contig_78:1275602-1271823	chromosome13_RaGOO:25001886-25002107	contig_808:3835369-3835148	chromosome13_RaGOO:18094847-18095068
bcar1	XM_017466321.1	scaffold_1355:2613936-2613526	chromosome9_RaGOO:31025257-31025667	scaffold_1463:3156621-3156211	chromosome9_RaGOO:30009167-30009577
bmpr1	XM_025053252.1	scaffold_1523:11318655-11324198	chromosome19_RaGOO:20701411-20705493	contig_296:5673788-5711714	chromosome19_RaGOO:28538346-28576952
DMRT1	MG765300.1	contig_231:4536517-4536741	chromosome15_RaGOO:5382294-5382449	contig_2141:774325-774558	chromosome15_RaGOO:4731595-4731828
DMRT3	MG765303.1	contig_231:4590408-4590854	chromosome15_RaGOO:5436101-5436547	contig_2141:830402-830827	chromosome15_RaGOO:4787672-4788097
gdf6	XM_015949386.1	contig_245:6034367-6033708	chromosome3_RaGOO:34704926-34705585	scaffold_1172:4452769-4453644	chromosome3_RaGOO:33048313-33049188
gsdf	AB525390.1	contig_51:13282480-13282349	chromosome8_RaGOO:20155510-20155379	contig_943:18559416-18559544	chromosome8_RaGOO:21154911-21155039
hsd17b	XM_023413720.1	scaffold_422:7021927-7022112	chromosome3_RaGOO:6167747-6167932	contig_1051:2675500-2675685	chromosome3_RaGOO:7549463-7549648
SOX3	AB775143.1	contig_68:18853052-18853765	chromosome2_RaGOO:43834501-43835214	contig_776:3090390-3091103	chromosome2_RaGOO:43462849-43463562
hsd11b	XM_015971584.2	scaffold_219:25085487-25085143	chromosome17_RaGOO:7683367-7683711	contig_970:481959-481666	chromosome17_RaGOO:5998641-5998934
foxl2	XM_015976261.2	contig_71:2050421-2051470	chromosome7_RaGOO:3834810-3835859	contig_260:2747402-2746404	chromosome7_RaGOO:3017382-3016384
foxl3	XM_015962648.2	contig_26:10446206-10445556	chromosome16_RaGOO:11860388-11861038	contig_4855:84153-84632	chromosome16_RaGOO:15024862-15025341
cyp19a1a	XM_015941214.2	contig_635:176842-176342	chromosome11_RaGOO29069181-29068681	contig_1259:2010988-2010479	chromosome11_RaGOO:30155180-30154680
rspo1	XM_015950063.2	scaffold_318:10679918-10680502	chromosome3_RaGOO:49155213-49154629	scaffold_1172:17915009-17914599	chromosome3_RaGOO:46510553-46510143
cyp11b	XM_015950577.2	scaffold_318:5417701-5418183	chromosome3_RaGOO-54417430-54416948	scaffold_1172:22354686-22354363	chromosome3_RaGOO:50950230-50949907
wnt4	XM_054734773.1	scaffold_35:2619687-2618869	chromosome14_RaGOO:1188709-1189527	contig_1443:1849270-1849926	chromosome14_RaGOO:2637461-2636805

Table S2. Primers for BAC clone verification.

BAC clone ID	Gene	Forward primer 5‘ – 3‘	Reverse primer 5‘ – 3‘
GRZ-B-a-201Bg09	*amha*	GTACCACGCCTTTCTTCTGC	GTGTTGCAAAAGCAGAACGG
GRZ-B-a-156Cc04	*amhr2*	TGGTGGCTGTGAAGGTGTAT	GTTTCTGTCCAGTGCCCAAG
GRZ-B-a-8Dd06	*bcar1*	CACCCTCTCCGTCATCTCTC	GTTCTACCAGGAGCAATGCG
GRZ-B-a-178Ah11	*DMRT3*	GAAGTGCATCCTGATCATCG	AGCTCCAGCTCCTCTGTCCT
GRZ-B-a-8Bg10	*gdf6*	TTACGCGCCTTGGTTTTGAA	GTGGATCAATCGTTCAGCCC
GRZ-B-a-201Bd03	*gdf6*	TTACGCGCCTTGGTTTTGAA	GTGGATCAATCGTTCAGCCC
GRZ-B-a-160Ac08	*gsdf*	CTCCAGGACCGAGAACTGTT	TTTCTGCACACACACACGAG
GRZ-B-a-242Cg02	*hsd17b*	TTTTGTGAGTGGGGTTCACA	GAGCAGGGCTGAAGTTGTTC
GRZ-B-a-170Bg04	*SOX3*	TCGAGCCAAAACAGAGAGGT	GGGAGAGGGGTCTTGATCTC

Table S3. General summary of statistics for *Fundulosoma thierryi* raw long and short reads used for genome assembly.

ONT raw reads *Fundulosoma thierryi* female	
Mean read length (bp)	19,165.7
Mean read quality	12.0
Median read length (bp)	13,717.0
Median read quality	12.2
Number of reads	2,895,020.0
Read length N50	33,246.0
STDEV read length	17,592.4
Total bases	55,485,189,972.0
Total coverage	66.93

Illumina raw reads *Fundulosoma thierryi* female	
Encoding	Sanger / Illumina 1.9
Total Sequences	902,296,328
Sequences flagged as poor quality	0
Sequence length	150
%GC	42
Total coverage	163.26

Table S4. General summary of statistics for *Nothobranchius guentheri* raw long-read data used for genome assembly.

PacBio raw reads *Nothobranchius guentheri* male	
Mean read length (bp)	22,672.6
Mean read quality	n/a*
Median read length (bp)	17,831
Median read quality	n/a*
Number of reads	4,853,133
Read length N50	37,456.0
STDEV read length	19,728.4
Total bases	110,032,915,262.0
Total coverage	148.09

*PacBio CLR data sequenced using the Sequel system do not contain quality information – the phred score is arbitrarily set to "!" (phred score = 0)

Table S5. *Fundulosoma thierryi* and *Nothobranchius guentheri* genome size estimation.

Fundulosoma thierryi female		
Property	Min	Max
Homozygous (aa)	99.8%	99.8%
Heterozygous (ab)	0.19%	0.19%
Genome Haploid Length	828,095,593 bp	829,018,997 bp
Genome Repeat Length	147,680,019 bp	147,844,696 bp
Genome Unique Length	680,415,574 bp	681,174,301 bp
Model Fit	80.86%	95.09%
Read Error Rate	0.21%	0.21%

Nothobranchius guentheri male		
Property	Min	Max
Homozygous (aa)	99.77%	99.75%
Heterozygous (ab)	0.23%	0.25%
Genome Haploid Length	741,793,291 bp	742,999,203 bp
Genome Repeat Length	218,247,327 bp	218,602,126 bp
Genome Unique Length	523,545,964 bp	524,397,078 bp
Model Fit	73.56%	98.3%
Read Error Rate	0.11%	0.11%

Table S6. Schematic depiction and means of the relative length and position of five chromosome markers mapped on chromosomes of *F. thierryi*.

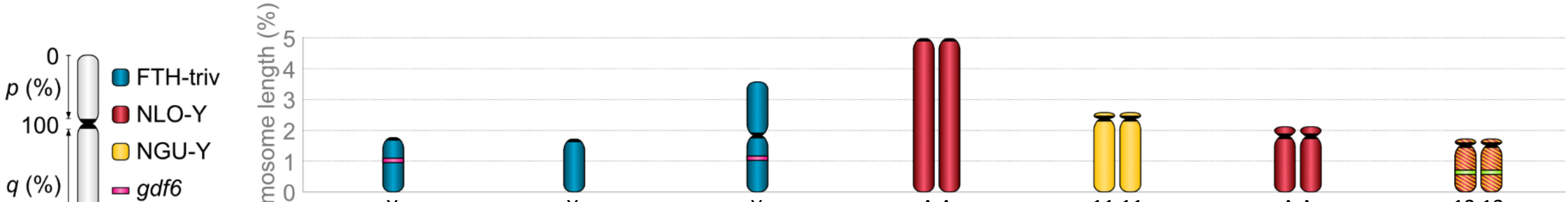

	X_1		X_2		Y		A A		11 11		A A		13 13	
	mean	SD	mean	SD	mean	SD	mean	SD	mean	SD	mean	SD	mean	SD
relative chromosome length (% of diploid karyotype)	1.74	0.01	1.72	0.02	3.58	0.02	4.98	0.05	2.57	0.06	2.13	0.09	1.70	0.07
p arm	0	0	0.37	0.30	48.56	0.31	0.02	0.05	7.24	2.04	14.97	3.45	10.60	2.78
q arm	100	0	99.63	0.30	51.44	0.31	99.98	0.05	92.76	2.04	85.03	3.45	89.40	2.78
FTH-triv														
distance	0	—	0	—	0	—	—	—	—	—	—	—	—	—
length	100	—	100	—	100	—	—	—	—	—	—	—	—	—
NLO-Y														
distance	—	—	—	—	—	—	0	—	—	—	0	—	0	—
length	—	—	—	—	—	—	100	—	—	—	100	—	100	—
NGU-Y														
distance	—	—	—	—	—	—	—	—	0	—	—	—	0	—
length	—	—	—	—	—	—	—	—	100	—	—	—	100	—
gdf6 (*q* arm)														
distance	57.27	5.29	—	—	59.57	4.34	—	—	—	—	—	—	—	—
amhr2 (*q* arm)														
distance	—	—	—	—	—	—	—	—	—	—	—	—	41.74	7.94

Mean values represent the relative (%) lengths and distances on the sex chromosomes and autosomes from three representative metaphases. Distance values: from the tip of the arm to the location of the marker. Length values: from the tip to the end of the marker region, the percentage reflects ratio between length of marker and length of the arm. SD – standard deviation. Dash means absence of a given marker on the chromosome or that the marker spans entire chromosome and was not measured. Where possible, we numbered the autosome pairs according to *N. furzeri* genome reference (Willemsen et al. 2020). For details, see main MS text.

Table S7. Schematic depiction and means of the relative length and position of five chromosome markers mapped on chromosomes of *N. furzeri*.

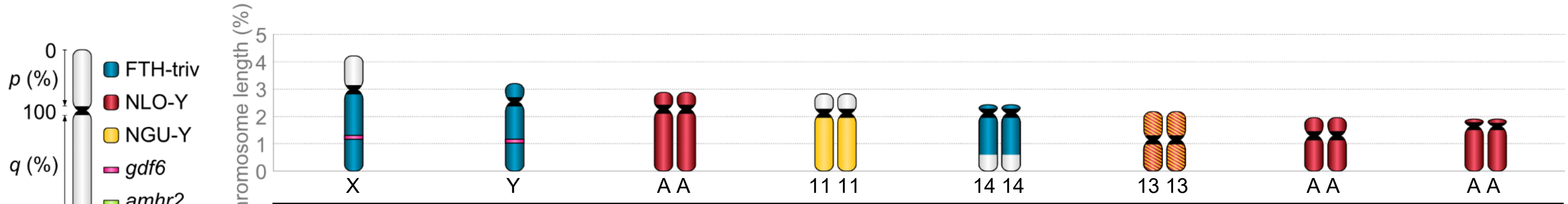

	X		Y		A A		11 11		14 14		13 13		A A		A A	
	mean	SD	mean	SD	mean	SD	mean	SD	mean	SD	mean	SD	mean	SD	mean	SD
relative chromosome length (% of diploid karyotype)	4.22	0.05	3.22	0.03	2.86	0.13	2.84	0.06	2.43	0.13	2.16	0.15	1.98	0.18	1.92	0.10
p arm	29.41	0.93	20.65	0.50	21.11	1.77	25.42	0.85	13.67	1.05	47.43	2.37	35.05	7.27	13.88	2.26
q arm	70.59	0.93	79.35	0.50	78.89	1.77	74.58	0.85	86.33	1.05	52.57	2.37	64.95	7.27	86.12	2.26
FTH-triv																
distance	*p* 0<br>*q* 0	—	*p* 0<br>*q* 0	—	—	—	—	—	*p* 0<br>*q* 29.77	—<br>2.46	—	—	—	—	—	—
length	*p* 0<br>*q* 100	—	*p* 0<br>*q* 100	—	—	—	—	—	*p* 100<br>*q* 70.23	—<br>2.46	—	—	—	—	—	—
NLO-Y																
distance	—	—	—	—	0	—	—	—	—	—	0	—	0	—	0	—
length	—	—	—	—	100	—	—	—	—	—	100	—	100	—	100	—
NGU-Y																
distance	—	—	—	—	—	—	*p* 0<br>*q* 0	—	—	—	0	—	—	—	—	—
length	—	—	—	—	—	—	*p* 0<br>*q* 100	—	—	—	100	—	—	—	—	—
gdf6 (*q* arm)																
distance	41.24	3.80	42.65	4.91	—	—	—	—	—	—	—	—	—	—	—	—
amhr2																
distance	—	—	—	—	—	—	—	—	—	—	—	—	—	—	—	—

Mean values represent the relative (%) lengths and distances on the sex chromosomes and autosomes from three representative metaphases. Distance values: from the tip of the arm to the location of the marker. Length values: from the tip to the end of the marker region, the percentage reflects ratio between length of marker and length of the arm. SD – standard deviation. Dash means absence of a given marker on the chromosome or that the marker spans entire chromosome and was not measured. Position of *amhr2* gene was not mapped. Where possible, we numbered the autosome pairs according to *N. furzeri* genome reference (Willemsen et al. 2020). For details, see main MS text.

Table S8. Schematic depiction and means of the relative length and position of five chromosome markers mapped on chromosomes of *N. lourensi*.

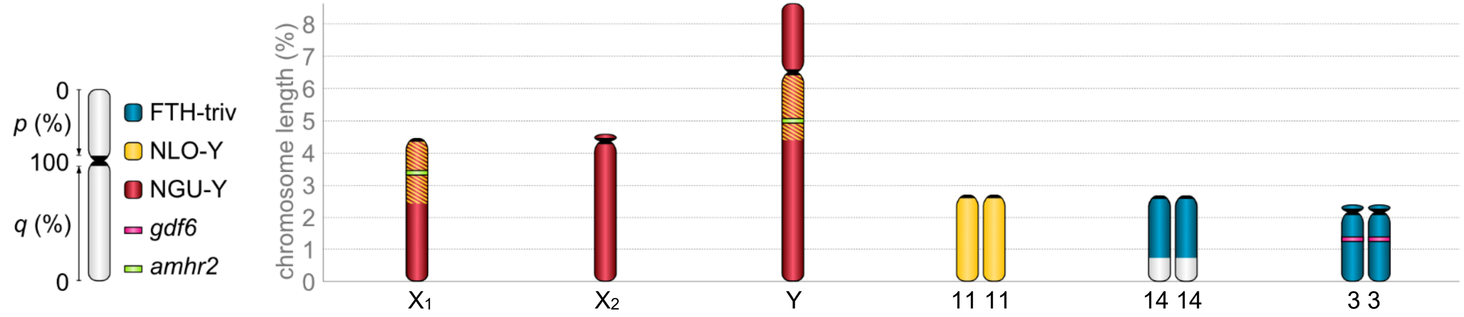

	X1 mean	X1 SD	X2 mean	X2 SD	Y mean	Y SD	11 11 mean	11 11 SD	14 14 mean	14 14 SD	3 3 mean	3 3 SD
relative chromosome length (% of diploid karyotype)	4.60	0.09	4.58	0.08	8.72	0.02	2.74	0.10	2.67	0.09	2.39	0.11
p arm	3.13	1.23	4.40	0.56	25.26	0.39	2.11	1.96	1.90	2.24	9.06	3.27
q arm	96.87	1.23	95.60	0.56	74.74	0.39	97.89	1.96	98.10	2.24	90.94	3.27
FTH-triv												
distance	—	—	—	—	—	—	—	—	*p* 0<br>*q* 27.80	—<br>3.75	0	—
length	—	—	—	—	—	—	—	—	*p* 100<br>*q* 72.20	—<br>3.75	100	—
NLO-Y												
distance	0	—	0	—	0	—	—	—	—	—	—	—
length	100	—	100	—	100	—	—	—	—	—	—	—
NGU-Y												
distance	*p* 0<br>*q* 54.77	—<br>3.08	—	—	*p* 0<br>*q* 67.25	—<br>1.56	0	—	—	—	—	—
length	*p* 100<br>*q* 45.23	—<br>3.08	—	—	*p* 0<br>*q* 32.75	—<br>1.56	100	—	—	—	—	—
gdf6 (*q* arm)												
distance	—	—	—	—	—	—	—	—	—	—	60.35	8.00
amhr2 (*q* arm)												
distance	76.17	2.99	—	—	77.14	2.44	—	—	—	—	—	—

Mean values represent the relative (%) lengths and distances on the sex chromosomes and autosomes from three representative metaphases. Distance values: from the tip of the arm to the location of the marker. Length values: from the tip to the end of the marker region, the percentage reflects ratio between length of the marker and length of the arm. SD – standard deviation. Dash means absence of a given marker on the chromosome or that the marker spans entire chromosome and was not measured. Where possible, we numbered the autosome pairs according to *N. furzeri* genome reference (Willemsen et al. 2020). For details, see main MS text. Autosomes carrying false-positive signals with *gdf6*-bearing BAC clone are not included.

Table S9. Schematic depiction and means of the relative length and position of five chromosome markers mapped on chromosomes of *N. guentheri*.

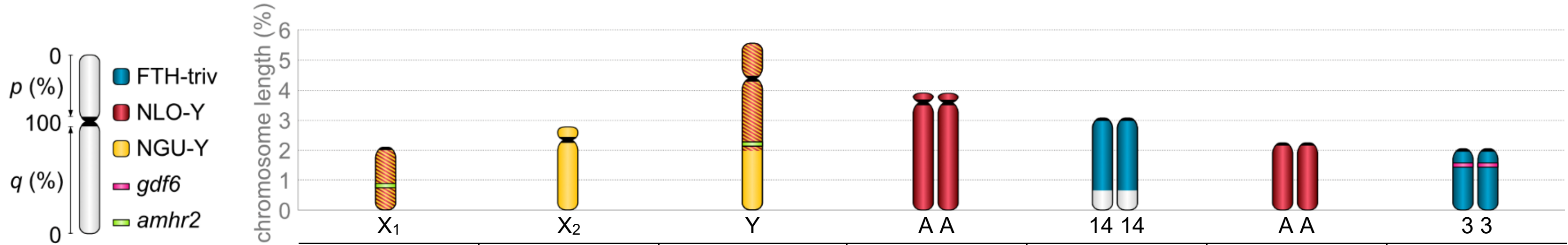

	X_1		X_2		Y		A A		14 14		A A		3 3	
	mean	SD	mean	SD	mean	SD	mean	SD	mean	SD	mean	SD	mean	SD
relative chromosome length (% of diploid karyotype)	2.09	0.04	2.78	0.04	5.56	0.09	3.91	0.23	3.06	0.09	2.28	0.03	2.04	0.06
p arm	0	—	15.80	0.94	21.13	0.59	7.94	1.42	0	—	1.52	2.37	0	—
q arm	100	—	84.20	0.94	78.87	0.59	92.06	1.42	100	—	98.48	2.37	100	—
FTH-triv														
distance	—	—	—	—	—	—	—	—	20.57	4.81	—	—	0	—
length	—	—	—	—	—	—	—	—	79.43	4.81	—	—	100	—
NLO-Y														
distance	0	—	—	—	*p* 0<br>*q* 45.00	—<br>4.42	0	—	—	—	0	—	—	—
length	100	—	—	—	*p* 100<br>*q* 55.00	—<br>4.42	100	—	—	—	100	—	—	—
NGU-Y														
distance	0	—	0	—	0	—	—	—	—	—	—	—	—	—
length	100	—	100	—	100	—	—	—	—	—	—	—	—	—
gdf6 (*q* arm)														
distance	—	—	—	—	—	—	—	—	—	—	—	—	73.34	4.47
amhr2 (*q* arm)														
distance	38.92	1.50	—	—	50.29	2.42	—	—	—	—	—	—	—	—

Mean values represent the relative (%) lengths and distances on the sex chromosomes and autosomes from three representative metaphases. Distance values: from the tip of the arm to the location of the marker. Length values: from the tip to the end of the marker region, the percentage reflects ratio between length of marker and length of the arm. SD – standard deviation. Dash means absence of a given marker on the chromosome or that the marker spans entire chromosome and was not measured. Where possible, we numbered the autosome pairs according to *N. furzeri* genome reference (Willemsen et al. 2020). For details, see main MS text.

Table S10. Schematic depiction and means of the relative length and position of five chromosome markers mapped on chromosomes of *N. brieni*.

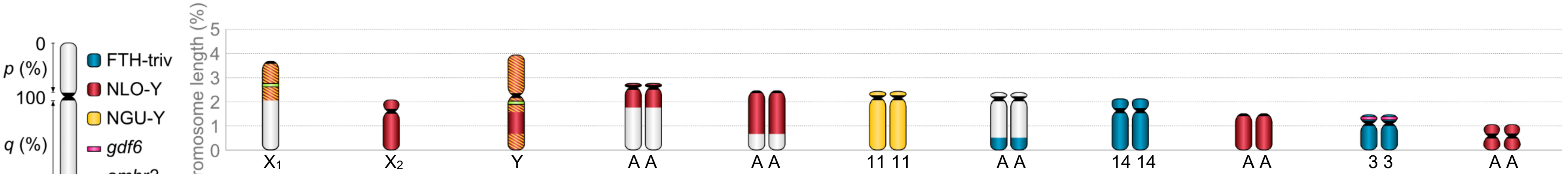

	X_1		X_2		Y		A A		A A		11 11		A A		14 14		A A		3 3		A A	
	mean	SD	mean	SD	mean	SD	mean	SD	mean	SD	mean	SD	mean	SD	mean	SD	mean	SD	mean	SD	mean	SD
relative chromosome length (% of karyotype)	3.59	0.05	2.37	0.02	3.93	0.03	2.77	0.07	2.46	0.03	2.42	0.07	2.36	0.02	2.13	0.03	1.50	0.07	1.44	0.16	1.03	0.02
p arm	0.65	0.92	25.49	0.22	42.51	0.67	6.27	0.44	0	0	11.26	2.83	26.91	1.80	23.51	1.38	0	0	24.22	2.84	45.16	2.4
q arm	99.35	0.92	74.51	0.22	57.49	0.67	93.73	0.44	100	0	88.74	2.83	73.09	1.80	76.49	1.38	100	0	75.79	2.84	54.84	2.47
FTH-triv																						
distance	—	—	—	—	—	—	—	—	—	—	—	—	0	0	*p* 0<br>*q* 0	—	—	—	0	—	—	—
length	—	—	—	—	—	—	—	—	—	—	—	—	27.87	3.23	*p* 0<br>*q* 100	—	—	—	100	—	—	—
NLO-Y																						
distance	*p* 0<br>*q* 57.18	—<br>4.34	0	—	0	—	*p* 0<br>*q* 67.86	—<br>1.83	26.88	1.68	—	—	—	—	—	—	0	—	—	—	0	—
length	*p* 100<br>*q* 42.82	—<br>4.34	100	—	100	—	*p* 100<br>*q* 32.14	—<br>1.83	73.12	1.68	—	—	—	—	—	—	100	—	—	—	100	—
NGU-Y																						
distance	*p* 0<br>*q* 42.82	—<br>4.34	—	—	*p* 0<br>*q* 0<br>*q* 69.06	—<br>—<br>5.41	—	—	—	—	0	—	—	—	—	—	—	—	—	—	—	—
length	*p* 100<br>*q* 57.18	—<br>4.34	—	—	*p* 100<br>*q* 29.77<br>*q* 30.94	—<br>3.13<br>5.41	—	—	—	—	100	—	—	—	—	—	—	—	—	—	—	—
gdf6 (*p* arm)																						
distance	—	—	—	—	—	—	—	—	—	—	—	—	—	—	—	—			71.45	3.20	—	—
amhr2 (*q* arm)																						
distance	75.68	2.86	—	—	86.92	0.79	—	—	—	—	—	—	—	—	—	—	—	—	—	—	—	—

Mean values represent the relative (%) lengths and distances on the sex chromosomes and autosomes (AA) from three representative metaphases. Distance values: from the tip of the arm to the location of the marker. Length values: from the tip to the end of the marker region, the percentage reflects ratio between the length of marker and length of the arm. Note two values for NGU-Y marker on the *q* arm of the Y chromosome reflecting two separated regions present on this arm. SD – standard deviation. Dash means absence of a given marker on the chromosome or that the marker spans entire chromosome and was not measured. Where possible, we numbered the autosome pairs according to *N. furzeri* genome reference (Willemsen et al. 2020). For details, see main MS text. Autosomes carrying false-positive signals with *gdf6*-bearing BAC clone are not included.

Table S11. Schematic depiction and means of the relative length and position of five chromosome markers mapped on chromosomes of *N. ditte*.

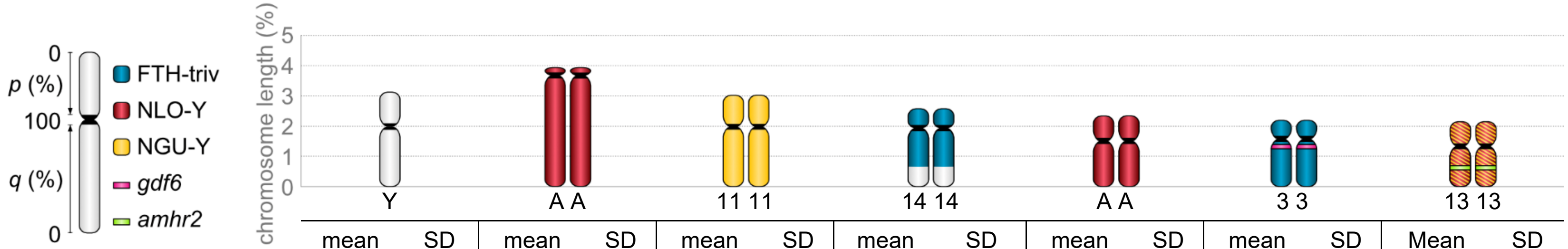

	Y		A A		11 11		14 14		A A		3 3		13 13	
	mean	SD	mean	SD	mean	SD	mean	SD	mean	SD	mean	SD	Mean	SD
relative chromosome length (% of diploid karyotype)	3.11	0.04	3.92	0.68	3.02	0.21	2.58	0.06	2.32	0.09	2.20	0.10	2.14	0.03
p arm	36.53	0.78	6.61	5.93	34.57	1.10	25.71	0.83	34.90	1.10	29.10	0.50	37.46	1.57
q arm	63.47	0.78	93.39	5.93	65.43	1.10	74.29	0.83	65.10	1.10	70.90	0.50	62.54	1.57
FTH-triv														
distance	—	—	—	—	—	—	*p* 0<br>*q* 30.40	—<br>3.99	—	—	0	—	—	—
length	—	—	—	—	—	—	*p* 100<br>*q* 69.60	—<br>3.99	—	—	100	—	—	—
NLO-Y														
distance	—	—	0	—	—	—	—	—	0	—	—	—	0	—
length	—	—	100	—	—	—	—	—	100	—	—	—	100	—
NGU-Y														
distance	—	—	—	—	0	—	—	—	—	—	—	—	0	—
length	—	—	—	—	100	—	—	—	—	—	—	—	100	—
gdf6 (*q* arm)														
distance	—	—	—	—	—	—	—	—	—	—	87.00	6.43	—	—
amhr2 (*q* arm)														
distance	—	—	—	—	—	—	—	—	—	—	—	—	46.89	6.20

Mean values represent the relative (%) lengths and distances on the Y chromosome and autosomes (AA) from three representative metaphases. Distance values: from the tip of the arm to the location of the marker. Length values: from the tip to the end of the marker region, the percentage reflects ratio between length of marker and length of the arm. SD – standard deviation. Dash means absence of a given marker on the chromosome or that the marker spans entire chromosome and was not measured. Where possible, we numbered the autosome pairs according to *N. furzeri* genome reference (Willemsen et al. 2020). For details, see main MS text.

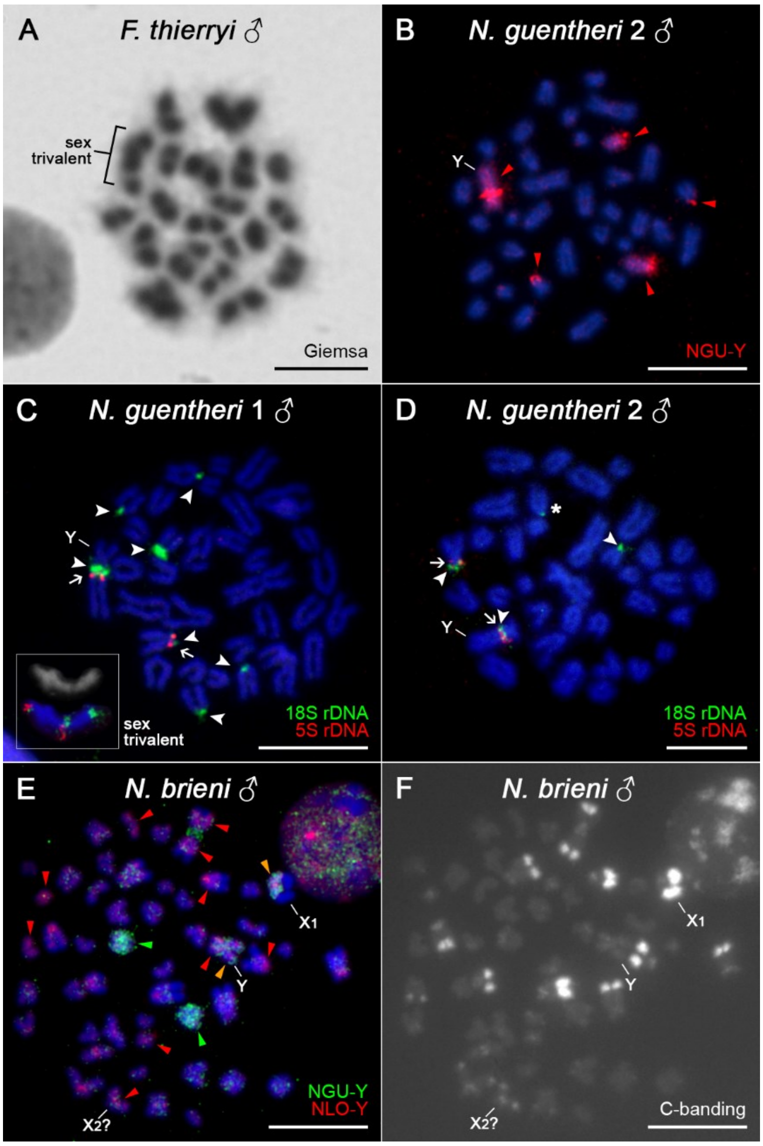

Figure S1. A collection of additional supportive cytogenetic results. **A**) An example of meiotic spread of *F. thierryi* male used for microdissection procedure. A detectable sex-trivalent which has been cut out for the painting probe construction is depicted. **B**) WCP with NGU-Y probe generated from NGU1 population on the male mitotic metaphase of NGU2 individual, confirming the identity of all three sex chromosomes also in this population. Note additional signals on the short arms of two acrocentric chromosomes. **C-D**) rDNA FISH hybridisation patterns in *N. guentheri* males of population NGU1 and NGU2, respectively. Dual-colour FISH with 5S (red signals; arrows) and 18S (green signals; arrowheads) rDNA probes. Chromosomes were counterstained with DAPI (blue). Note that 18S rDNA clusters occupy short arms of six acrocentric or subtelocentric chromosomes (including X chromosomes), and interstitial region on the long arms of Y chromosome in NGU1 population, while they are restricted to three sex chromosomes in NGU2 population. Asterisk points on additional putative 18S rDNA signal which, however, has not been observed on overwhelming majority of analysed metaphase spreads (in total 60) in this individual and other two analysed NGU2 males. In the population NGU1 (**C**), we probed also meiotic plates and a trivalent formed from X_1X_2Y sex chromosomes is herein framed, along with rDNA signal patterns confirming that all three members of the system carry one or both rDNA clusters. 5S rDNA probe showed, in contrast to the 18S rDNA one, the same patterns in both populations, as it overlaps with the 18S rDNA cluster on the Y

chromosome and one X chromosome. (**E**, **F**) *N. brieni* male metaphase after sequential cytogenetic analysis. **E**) Zoo-FISH with NGU-Y (green arrowheads) and NLO-Y (red arrowheads) probes delimiting clearly the identity of the Y and X_1 sex chromosome (overlapping signals; orange arrowheads). The NLO-Y probe produced signals over majority of *N. brieni* chromosomes, hence, those marked by red arrowheads belong to a subset showing consistent pattern of brighter signals covering majority of the chromosome length across studied metaphases. **F**) C-banding (with DAPI counterstaining) performed on the same metaphase to delimit the identity of X_2 sex chromosome and whether it belongs to the set of chromosomes stained by NLO-Y probe. In line with our previous C-banding analyses in males and females of the same population of *N. brieni* (Lukšíková et al. 2023) we inferred the most probable candidate for X_2 sex chromosome. Scale bar = 10 μm.

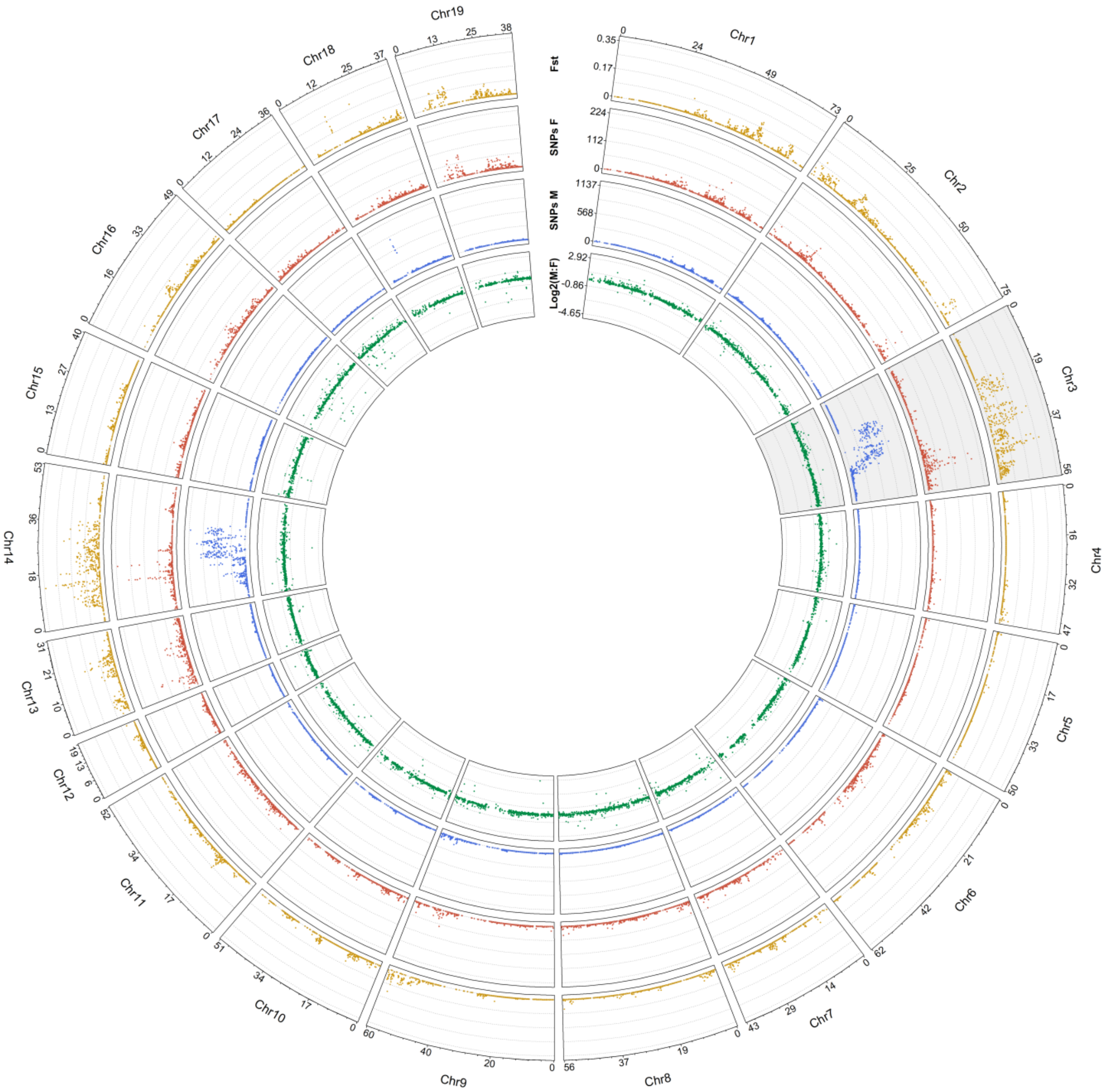

Figure S2. Identification of sex-linked regions in *F. thierryi*. Circo plots show results of coverage (inner green ring) and Pool-seq analyses (male and female specific SNPs and relative divergence (F_{ST}) are in blue, red and yellow, respectively) using pseudochromosomes constructed with *N. furzeri* as a reference (Willemsen et al. 2020). The sex chromosome of *N. furzeri* is shaded in gray.

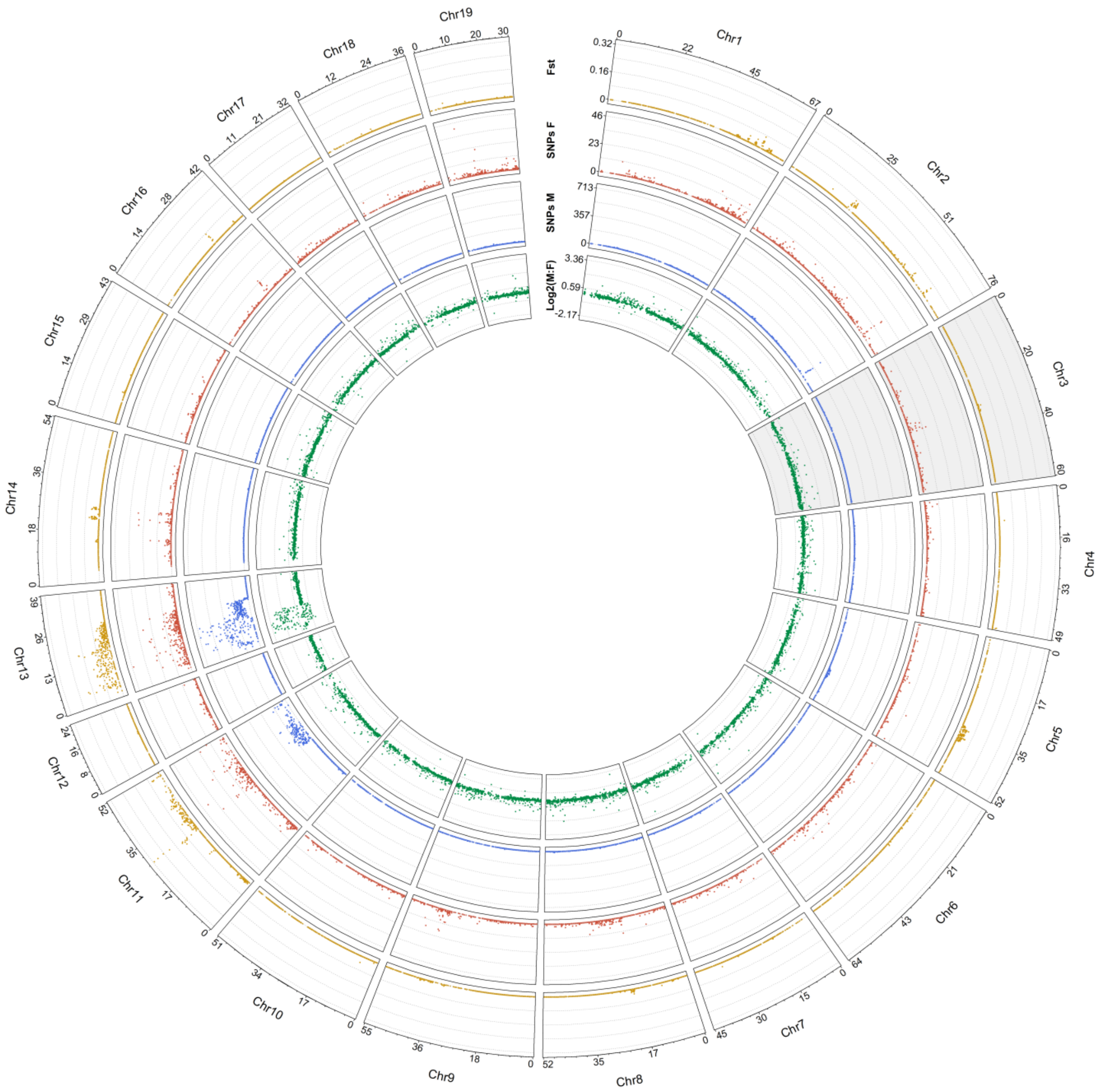

Figure S3. Identification of sex-linked regions in *N. guentheri*. Circo plots show results of coverage (inner green ring) and Pool-seq analyses (male and female specific SNPs and relative divergence (F_{ST}) are in blue, red and yellow, respectively) using pseudochromosomes constructed with *N. furzeri* as a reference (Willemsen et al. 2020). The sex chromosome of *N. furzeri* is shaded in gray.

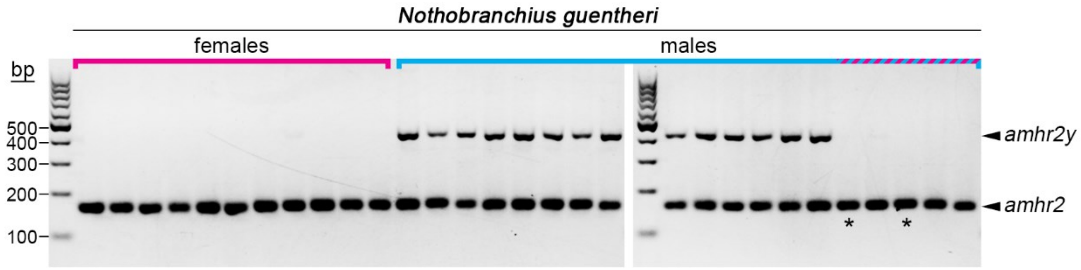

Figure S4. Agarose gel electrophoresis of duplex PCR of *amhr2* alleles in *Nothobranchius guentheri* males and females. The T>C substitution in the exon 5 of the *N. guentheri amhr2* Y-linked allele was verified using two sets of primers, each amplifying different regions of the *amhr2* alleles. The segment common to both X and Y *amhr2* alleles (expected fragment size: 156 bp) is designated as *amhr2*. The amplicon specific for Y-chromosomal allele (expected fragment size: 411 bp) is designated as *amhr2y*. The expected output of duplex PCR was therefore a single amplicon in females and two amplicons in males. We performed the analysis on individuals from both our *N. guentheri* populations, NGU1 (N = 15 males, 11 females from the population NGU1) and NGU2 (N = 4 males). The duplex PCR assay confirmed sex in 15 NGU1 males and all four tested males from the population NGU2. However, note that five NGU1 males (crosshatched blue-violet line) lacked the Y-specific amplicon. All 11 females had only a single PCR band as expected. The PCR products were loaded on a 2% agarose/1xTBE gel stained with GelRed™ Nucleic Acid Gel Stain (Biotium, Hayward, CA, USA), run at 60 V for 150 min, along with 2 µl GeneRuler™ 100 bp DNA Ladder (Biogen Life Technologies, Prague, Czech Republic).

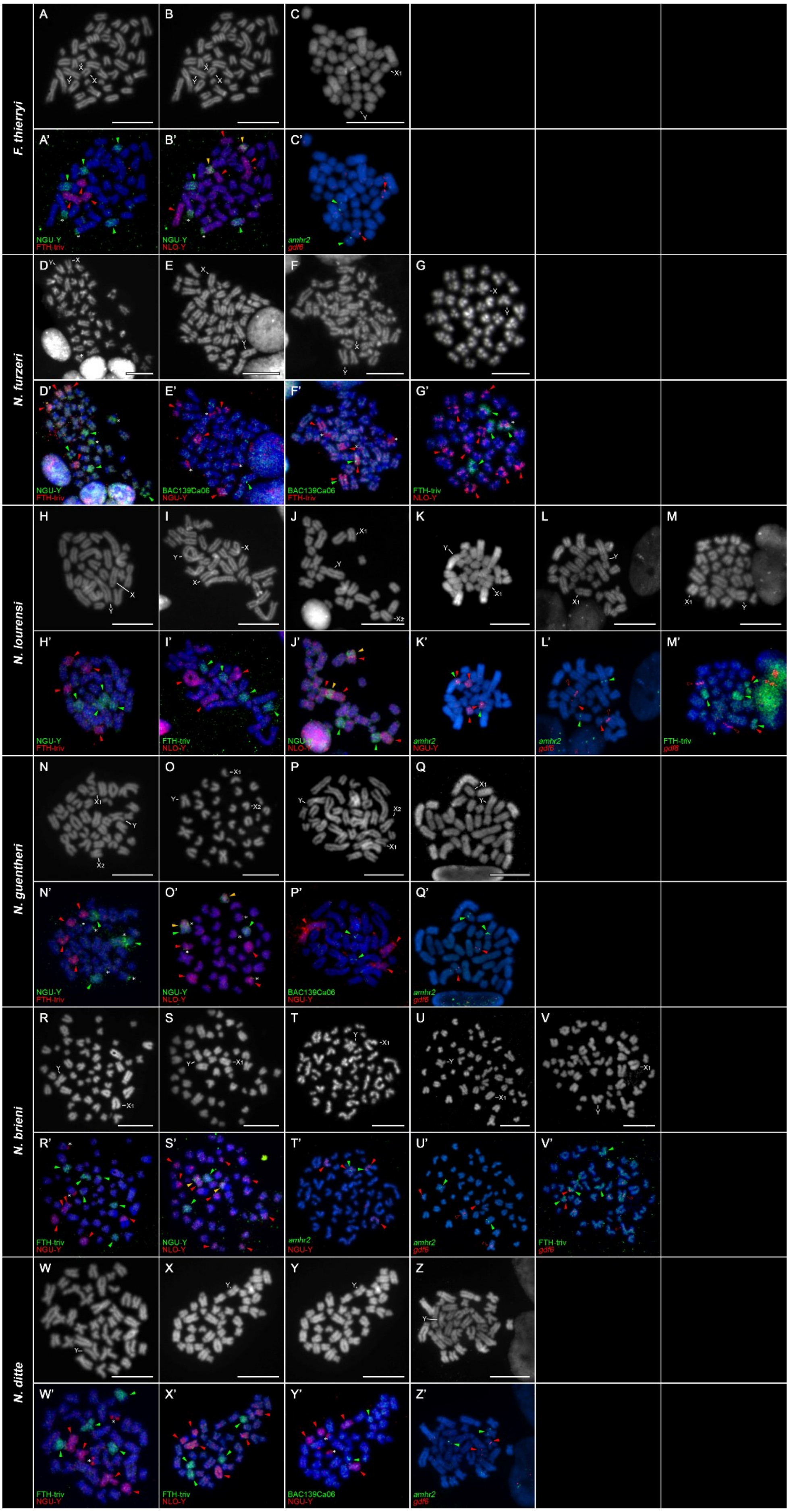
F. thierryi
N. furzeri
N. lourensi
N. guentheri
N. brieni
N. ditte
NGU-Y
FTH-triv
NLO-Y
amhr2
gdf6
BAC139Ca06

Figure S5. Male mitotic metaphases of studied *Nothobranchius* and *Fundulosoma* species after cross-species experiments with WCP and BAC-FISH probes. To improve clarity of the signal patterns, some included metaphases were reprobed for two different experiments. For each species, upper row represents DAPI images, converted to grayscale, while bottom row shows merged FISH images with two mapped fluorescent probes (green and red signals; arrowheads) and DAPI (blue). Sex chromosome-derived probes: NGU-Y (*N. guentheri*; Y chromosome), NLO-Y (*N. lourensi*; Y chromosome), FTH-triv (*F. thierryi*, whole meiotic sex-trivalent X_1X_2Y). BAC-FISH: clones derived from *N. furzeri* BAC library carrying *gdf6* and *amhr2* gene, and Y-linked 139Ca06 clone (Reichwald et al. 2015). Sex chromosomes are marked if possible (see upper DAPI images). X chromosomes are distinguished as X_1 (ancestral) or X_2 (new autosome addition; neo-X) if the signal patterns allow that. Asterisks denote additional signals which, based on the complementary data, represent major ribosomal DNA (rDNA) sites whose tandem arrays are located also on the Y of *N. guentheri* (hence being included in the painting probe). Note that painting probes fully stain all members of the X_1X_2Y system from which they were derived (in *F. thierryi*, *N. guentheri*, *N lourensi*). In all analysed species, NGU-Y and NLO-Y probes partially overlap (orange arrowheads) in one segment (*N. guentheri*, *N. lourensi*, *N. brieni*) or by covering entire autosome pair (*F. thierryi*), or its short arms (*N. ditte*). The Y-linked regions stained both by NGU-Y and NLO-Y probes also carry *amhr2* BAC-FISH signal. Notice also that *F. thierryi* and *N. furzeri* share the synteny block carrying *gdf6* gene (on the long arms of the Y chromosome in *N. furzeri*, and close to centromere on the Y of *N. lourensi*). In *N. ditte*, none of the mapped probes produced signals on the Y. In *N. furzeri* (G'), note that NLO-Y probe generated eight distinctive signals together with a number of additional putative signals (including the one on the Y chromosome, denoted by asterisk) which may reflect shared repetitive DNA content (Štundlová et al. 2022; Lukšíková et al. 2023). In *N. lourensi* (L', M') and *N. brieni* (U', V'), we observed four signals after BAC-FISH with the clone containing *gdf6* gene. Reprobing with FTH-triv painting probe allowed us to identify which pair of signals reside on the synteny blocks harboring the original *gdf6* locus (full red arrowheads). The remaining pair of signals (empty red arrowheads) might represent false positives due to repetitive sequences contained on the selected BAC clone. Scale bar = 10 μm.

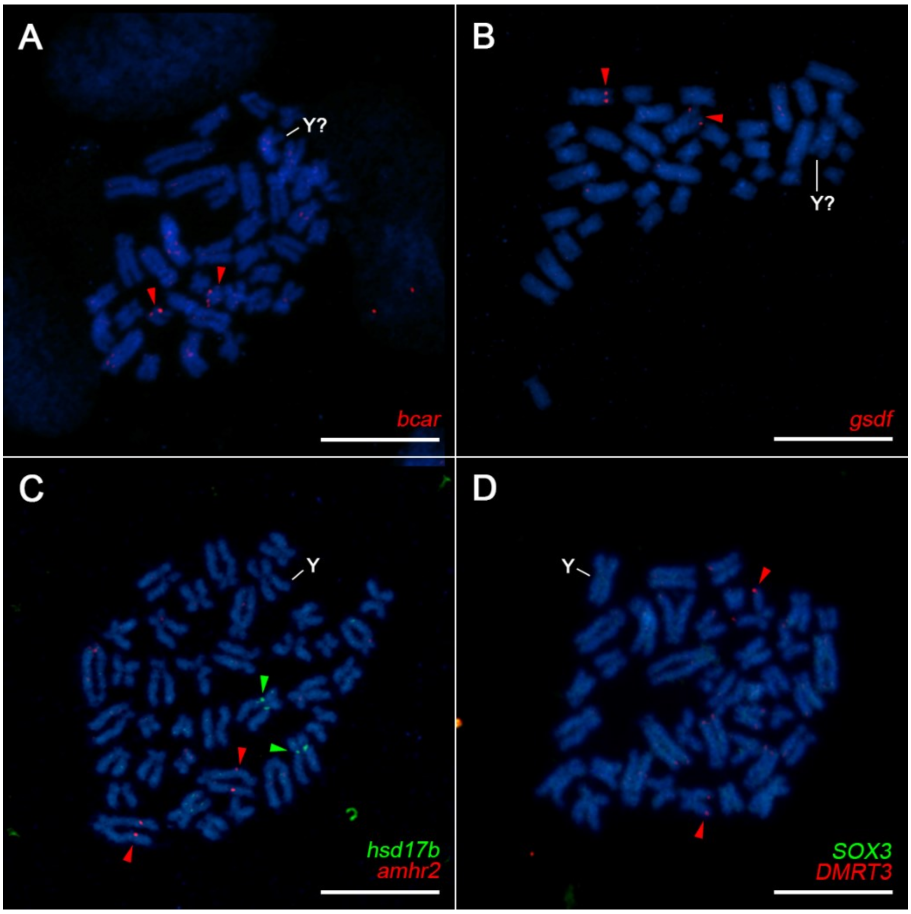

Figure S6. Male mitotic metaphases of *Nothobranchius ditte* after BAC-FISH with carrying common candidate genes for the sex-determining role in teleosts. Clones are derived from *N. furzeri* BAC library (Reichwald et al. 2015) and their detailed description is provided in Table 2 (main MS text) and Table S1. Note that except for *SOX3* (**D**) and *amha* (not shown) we obtained reproducible signals (together with variable degree of non-specific hybridisation signals across different metaphases) for all other tested genes (*gdf6* shown in Figure S5). None of the mapped BACs generated consistently odd signal patterns which could suggest the location of a given gene on X_1X_2Y multiple sex chromosomes. The Y chromosome is indicated (with a varied degree of certainty) based on its exclusive size and morphology (i.e. the only medium-sized metacentric chromosome in the complement). Scale bar = 10 μm.

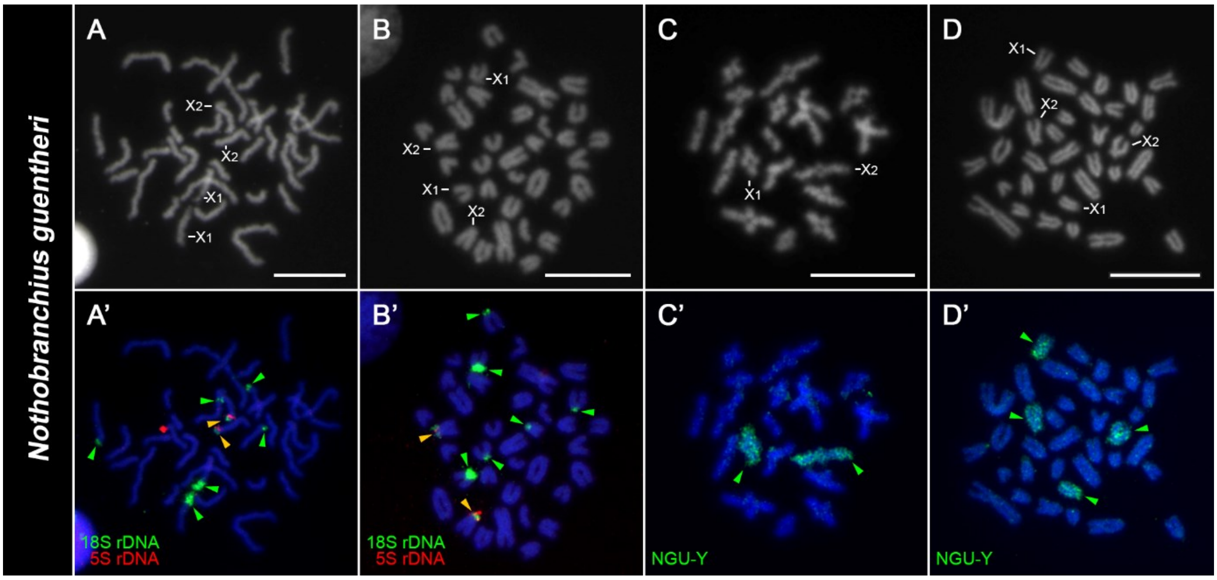

Figure S7. Mitotic and meiotic spreads of two *Nothobranchius guentheri amhry*- males after WCP with NGU-Y probe and 5S/18S rDNA FISH. (**A**) mitotic prometaphase; (**B**, **D**) mitotic metaphase; (**C**) meiotic prophase I (diplotene/diakinesis). For each individual, upper row represents DAPI images, converted to grayscale, while bottom row shows merged FISH pictures with two mapped fluorescent probes (green and red signals; arrowheads) and DAPI (blue). Sex chromosomes are marked if possible (see upper DAPI images). (**A'**, **B'**) Dual-colour FISH with 5S (red signals; orange arrows) and 18S (green signals; green or orange arrowheads) rDNA probes. Orange arrowheads represent co-localisation/adjacent arrangement or 5S and 18S rDNA cluster and reside on *N. guentheri* sex chromosomes (compare with Figure S1C, D). (**C'**, **D'**) NGU-Y = painting probe derived from *N. guentheri* Y sex chromosome (green signals and arrowheads). Chromosomes were counterstained with DAPI (blue). In both male individuals, we observed a female constitution with 2n = 36 and two pairs of sex chromosomes, most likely corresponding to $X_1X_1X_2X_2$ sex chromosome constitution. Chromosomes in meiotic prophase I (C) were paired in 18 standard bivalents and instead of typical male sex-trivalent (compare with Figure S1C) four chromosomes painted by the NGU-Y probe were observed (C'). Scale bar = 10 μm.

All herein cited references are cited also in the main MS text.
